# Imported malaria definition and minimum data for surveillance

**DOI:** 10.1038/s41598-022-22590-6

**Published:** 2022-10-26

**Authors:** Nicholas J. Arisco, Cassio Peterka, Marcia C. Castro

**Affiliations:** 1 Department of Global Health and Population, Harvard T.H. Chan School of Public Health, Boston, MA USA; 2 Secretaria de Vigilância em Saúde, Ministério da Saúde, Brasília, DF Brazil

**Keywords:** Public health, Epidemiology

## Abstract

The mobility of malaria-infected individuals poses challenges to elimination campaigns by way of spreading parasite drug resistance, straining country-to-country collaboration, and making routine data collection difficult, especially in resource-poor settings. Nevertheless, no concerted effort has been made to develop a common framework to define the spatial and temporal components of an imported malaria case and recommend the minimum data needed to identify it. We conducted a scoping review of imported malaria literature from 2010 to 2020 which showed that definitions vary widely, and local capabilities of detecting importation are often restricted in low-income countries. Following this, we propose a common definition for imported malaria and the minimum data required to identify a case, depending on the country’s capability of conducting an epidemiological investigation. Lastly, we utilize the proposed definition using data from Brazil to demonstrate both the feasibility and the importance of tracking imported cases. The case of Brazil highlights the capabilities of regular surveillance systems to monitor importation, but also the need to regularly use these data for informing local responses. Supporting countries to use regularly collected data and adopt a common definition is paramount to tackling the importation of malaria cases and achieving elimination goals set forth by the World Health Organization.

## Introduction

Imported malaria consistently challenges elimination efforts globally^1,2^. Mobility of infected individuals has resulted in disease reintroduction after elimination^3,4^, has contributed to the spread of drug-resistant parasites within and between countries^5–8^, and has strained relationships between neighboring countries with distinct control strategies^9–11^. The World Health Organization (WHO)’s Global Technical Strategy (GTS) for Malaria aims to reduce transmission of the disease by 90% by 2030 with major goals set for elimination^12^. However, globalization and increased connectedness continue to alter the elimination playing field^13^, and imported malaria is specifically cited as a unique and complex challenge to achieving those goals^3,14–16^.

Combatting imported malaria presents key challenges. Country-specific surveillance systems’ ability to diagnose and collect data necessary to determine importation status varies^17,18^. Many countries lack the capacity to distinguish an autochthonous from an imported case^12^, which may hamper cross-country collaboration in data collection (a paramount action in the battle against imported malaria)^19^. Furthermore, low-resource settings are challenged by the unfeasibility of scaling molecular techniques necessary for tracking the introduction of drug-resistant parasites^20^, with potential consequences for the deployment of effective treatment. Inadequate surveillance is further challenged by the rate of mobility of infected individuals, which can change rapidly in response to economic, political, climatological, or other stressors^21,22^. A country’s ability to diagnose, identify, and ultimately treat imported malaria cases hinges upon robust surveillance and treatment systems capable of managing large and sudden influxes of malaria cases.

The WHO defines imported malaria as a “malaria case or infection in which the infection was acquired outside the area in which it is diagnosed” provided diagnosis is within three months of returning from an endemic area^23^. However, many countries have adopted unique methods for defining imported malaria at different temporal and spatial scales best suited to their surveillance capabilities, local malaria epidemiology, and geographical position in relation to other malaria-endemic countries^2^. Also, data collection and surveillance systems used to identify imported malaria cases vary dramatically in their capabilities from country to country^17,24^, with certain countries having a high capacity to identify and track cases while even close neighbors do not^17^. The framework used to identify imported malaria cases may influence a country’s response. For example, in countries nearing elimination, if imported cases are only classified by international movement, and not between endemic and non-endemic regions within a country, mobility-centric interventions like case detection on travel routes are likely to be focused on major international ports of entry/border regions rather than hotspots for local movement within the country. The failure to recognize within-country mobility overlooks the importance of local receptivity and vulnerability in the malaria elimination process^25^.

Given the stress that imported malaria imparts on elimination efforts, it is important to use a common conceptual framework to define imported cases, considering the spatial and temporal scales at which importation occurs. This is necessary common ground on which results in academic publications can be compared accurately, and cross-country collaboration in surveillance and control can be built. Yet, such a framework is not available. Here we address this gap. We review definitions and data sources for imported malaria reported in the literature between 2010 and 2020. Next, based on our review, we propose a conceptual framework and minimum data needed to define imported cases and to compute spatial and temporal trends of imported malaria as countries approach elimination. Finally, we leverage data from the Brazilian Malaria Epidemiological Surveillance Information System (SIVEP) to apply the proposed conceptual framework and provide a country example of the efficacy of national surveillance in capturing and identifying the imported malaria burden. The analysis of these data offers context to the proposed conceptual framework, provides a comprehensive example of the potential of a national surveillance system in identifying imported malaria, and identifies the minimum necessary data for countries to track importation.

## Methods

### Scoping review: search strategy and selection criteria

A scoping review was conducted of available articles on malaria importation published between January 1st, 2010, and December 1st, 2020, from PubMed (https://ncbi.nlm.nih.gov/pubmed/), EMBASE (www.embase.com), and Web of Science (www.webofknowledge.com). Articles in PubMed were searched for using the following query: (((Imported[Title/Abstract] OR non-endemic[Title/Abstract] OR formerly endemic[Title/Abstract]) AND (“imported malaria”[Title/Abstract])) AND (malaria OR malaria[MeSH] OR malaria[Title/Abstract] OR “malaria s”[Title/Abstract] OR malarias[Title/Abstract] OR malariae[Title/Abstract])). Articles in Web of Science were searched for using the following query: ((((TI = (imported) OR AB = (Imported)) OR (TI = (non-endemic) OR AB = (non-endemic)) OR (TI = (formerly endemic) OR AB = (formerly endemic))) AND (TI = (imported malaria) OR AB = (imported malaria))) AND (ALL = (malaria) OR TS = (malaria) OR (TI = (malaria) OR AB = (malaria)) OR (TI = (malaria s) OR AB = (malaria s)) OR (TI = (malarial) OR AB = (malarial)) OR (TI = (malariae) OR AB = (malariae)))). Articles in Embase were searched for using the following query: ((('imported':ab,ti OR 'non-endemic':ab,ti OR 'formerly endemic':ab,ti) AND ('imported malaria':ab,ti)) AND ('malaria' OR 'malaria':ab,ti OR 'malaria s':ab,ti OR 'malarias':ab,ti OR 'malariae':ab,ti)).

Articles in English were included in this analysis, and duplicates were screened for upon first pass. Search results were then deemed relevant to this analysis by reading of title and abstract first, followed by a full text review of those relevant. Criteria for exclusion included language other than English, studies that did not focus on human malaria, analysis of treatment protocols, drug efficacy studies, documentation of new screening tools, studies focusing on diseases other than malaria, studies on the cost-effectiveness of interventions/treatment, studies on solely disease vectors, analysis of chemoprophylaxis, program evaluation studies not related to malaria, articles that did not mention imported cases in their title or abstract, articles that had to do with diagnostic techniques, and articles solely focused on autochthonous/aboriginal malaria. If an article did not have an explicitly stated definition of imported malaria, or if an article did not contain information/data amendable to determining a definition, it was excluded from the final analysis.

**Figure 1:**
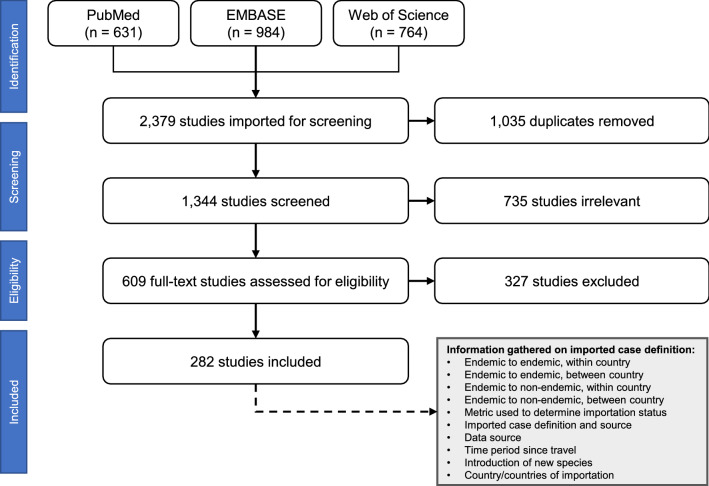
Flow diagram of scoping review.

Each article was classified by its explicit or implicit definition of imported malaria, the country of focus for importation, and the data source used to determine importation status (Fig. 1). In addition to the articles gleaned from the scoping review, major multinational case reporting documentation was analysed, including the WHO World Malaria Report and reports from the European Centre for Disease Prevention and Control.

### Description of surveillance system data for Brazil case study

Brazil has a vast network of laboratories and clinics across the country that can diagnose malaria to the species level and treat infections free of charge. In Brazil, all cases are confirmed by microscopy or a rapid test (no case is reported based solely on clinical manifestations). When individuals test positive for malaria, the technician records demographic information, travel history, and other characteristics from the individual, and this information is then entered into SIVEP. For this study, de-identified, individual-level data on reported malaria cases in Brazil were obtained from SIVEP for the years 2007–2018. The following variables were extracted: date of notification; parasite type; municipality where the case was reported; municipality/country where the infection was likely to have occurred, deduced by epidemiological investigation; date of the first symptoms; and municipality/country of residence.

### Ethics approval

This study used de-identified data and is exempted from human subjects’ review.

## Results

### Imported malaria in summary reports

The 2020 World Malaria Report (WMR)^26^ compiles comprehensive data from country-level surveillance systems and reported that 45 of the 103 malaria endemic countries did not collect any data on imported malaria cases between 2000 and 2019. 35 of those countries were in sub-Saharan Africa, and all are still high-burden countries. Ten countries were denoted as reporting annual data while 54 countries had incomplete data. Non-reporting highlights a need for heightened surveillance to capture imported malaria cases in endemic countries. The Global Malaria Programme (GMP) proposes a framework for the classification of malaria cases which groups cases into either imported, induced, or indigenous^27^. Specifically, imported cases are defined as “[A case] that is due to mosquito-borne transmission and is acquired outside the area in which it was detected, in a known malarious area to or from which the patient has travelled outside the elimination area” with specific timeframes designated based on infectiousness periods of each parasite.

In the European Union, a network of surveillance systems exists through the European Centre for Disease Prevention and Control (ECDC) called The European Surveillance System (TESSy). TESSy requires all EU member states as well as Iceland, Liechtenstein, and Norway to report data from their country-specific surveillance systems and defines cases and required case information in line with the European Commission on communicable disease case definitions developed between 2008 and 2012^28^. The European Commission does not specifically outline an imported case definition, but rather categorizes cases as possible, probable, and confirmed; travel history is recorded for each case to determine where the case may have originated. In 2018, of the 8347 confirmed malaria cases, 7338 had a known importation status^28^.

### Imported malaria in the literature

Our scoping review resulted in 2379 articles (Fig. 1). After removal of 1035 duplicated and 735 articles deemed irrelevant, 609 articles were fully reviewed. Of those, 327 were excluded based on the ability of the article to provide either a definition of imported malaria or the ability to deduce aspects of a definition based on the information provided in the article. A total of 282 articles were included in the analysis (Supplementary Table S1). Of the 282 articles included in the review, 97 (34.4%) explicitly stated imported malaria cases definitions (Table 1). The remaining 186 studies contained enough information to determine how the authors were defining an imported malaria case (for example, if a paper assessed importation in a non-endemic country and did not provide a definition, then all cases were assumed to be imported from endemic countries, or if a study referred to both within-country and between-country movement as imported cases, then the implicit definition would include both these movement types). The most common non-country-specific definition cited was that of the WHO (n = 33, or 34.0% of papers with explicit definitions)^29^, and it provided the foundation upon which our proposed framework was developed. The most used country-specific definition cited was that of the Technical Scheme for Malaria Elimination in China (n = 33, or 34.0% of papers with explicit definitions). China defines an imported case as “a malaria case who travelled to any malaria-endemic areas outside China within the month before illness onset, and the last country visited with ongoing malaria transmission was taken as the potential location of infection”^30^ (a period three times shorter than that proposed by the WHO). Other countries/regions that were found to have explicit definitions for imported malaria specifically referred to in the literature were the United States, Brazil, Reunion, Swaziland, Malaysia, Sri Lanka, South Africa, and the Philippines.

**Table 1:** Summary of scoping review on definitions of imported malaria.

Definition Sources (number of studies using definition)	Imported malaria case definition	Spatial level considered in definition				Information on time of travel	Country in study receiving cases
		Local importation		Cross-border			
		Within units	Between units	Border	Trans-national		
**Explicit definition included in study text (n = 97; 34.4% of studies)**							
World Health Organization^62^ (**n = 33**^**†**^)	A case acquired outside the country. “The origin of imported cases can be traced to a known malarious area outside the country to which the case has travelled. In areas with ongoing local transmission, elimination programmes should reserve the category ‘imported’ for ‘exotic’ parasite species and very recent arrivals from endemic countries (within the past 3 months). For all other cases occurring during the transmission season, it is prudent to assume a local origin of the infection.”			✓	✓	**All studies:** 3 months	European Union, France, Qatar, Cape Verde, Canada, China, Portugal, Sri Lanka, United States, Japan, Brazil, Saudi Arabia, United Arab Emirates, South Africa, Belgium, Portugal, Oman, Nepal, Iran, Bhutan, Italy
Chinese Center for Disease Control and Prevention^63^ (**n = 33**^**†**^) Academic articles (**n = 6**^**†**^)	A case acquired in an endemic country or endemic administrative area within the country and diagnosed in a non-endemic country/region, variable time frame for travel	✓	✓	✓	✓	**China:** 1 month **South Africa**: 1 month **Other countries**: None	China, Bhutan, Nepal, Malaysia, Republic of Korea, South Africa, Mayotte
United States Centers for Disease Control and Prevention Malaria Surveillance Report^64^ (**n = 1**^†^) European Centre for Disease Prevention and Control^65^ (**n = 1**^†^) Academic articles (**n = 21**^†^)	A case acquired in an endemic country and diagnosed in a non-endemic country, variable time frame for travel			✓	✓	**Namibia:** 2 weeks **Suriname:** 2 weeks **Other countries:** None	United States, Réunion, European Countries, South Africa, Namibia, Suriname,
Academic articles (**n = 2**^**†**^; S1 Table)	A case acquired in an endemic administrative area and diagnosed in a different administrative area, no time frame for travel	✓	✓			**All studies:** None	Brazil, Madagascar
**Information discussed in studies with no explicit definition (n = 185; 65.6% of studies)**							
Academic articles (**n = 73**^**†**^) *Note: *All from perspective of non-endemic countries*	All cases diagnosed in country, variable time frame for travel			✓	✓	**United Kingdom:** 12 months **Italy:** 6 months **Australia:** 1 month **Other countries:** None	France, Serbia, United States, Italy, Spain, Romania, Turkey, Russia, United Kingdom, New Zealand, Taiwan, Puerto Rico, Canada, Netherlands, Sri Lanka, Portugal, Australia, Finland, JapanAlbania, Denmark, Poland, Slovak Republic, Scotland, China, Sweden, Germany, Belgium, Norway, Switzerland
Multiple studies (**n = 92**^**†**^)	No explicit spatial component^‡^ of definition was provided in the study, time frame for travel variable			✓	✓	**Spain:** 1 month / 6 months **Brazil:** 1 month **Eswatini:** 2 weeks **United Kingdom:** 1 week **China:** 1 month **Other countries:** None	Spain, Qatar, Italy, United Kingdom, Iran, Malaysia, France, Guatemala, South Korea, China, United States, Cabo Verde, Sri Lanka, Nepal, Puerto Rico, Swaziland, Suriname, Canada, Saudi Arabia, Chile, Germany, Jordan, Venezuela, Bangladesh, Japan, Poland, New Zealand, South Africa, Greece, Denmark, Reunion, Bulgaria, Botswana, Namibia, Eswatini, Thailand
Multiple studies (**n = 6**^**†**^)	No explicit spatial component^‡^ of definition was provided in the study, time frame for travel variable	✓	✓			None listed	United Kingdom, India, Ethiopia, Colombia Kenya, Bangladesh
Multiple studies (**n = 14**^**†**^)	No explicit spatial component^‡^ of definition was provided in the study, time frame for travel variable	✓	✓	✓	✓	**Brazil:** 1 month **Other countries:** None	Brazil, Saudi Arabia, Eswatini, China, Iran, Myanmar, Zanzibar

^†^Elaborated on and cited in S1 Table.

^‡^Spatial levels that were discussed in the study are recorded in the table despite a formal definition being put forth to include these levels.

Since specific spatial designations were omitted in the current WHO definition, two spatial levels of importation were gleaned from the articles included in the analysis: country to country (referred to as cross-border malaria), and within-country importation. Cross-border malaria can further be broken down into transmission at/across the border (referred to as border malaria) and country to country not at the border (referred to as transnational malaria). Internal mobility can be broken down by administrative levels, based on governing jurisdiction. Countries are generally divided into second- (states, provinces, prefectures, and others) and third-level administrative units (communes, counties, municipalities, districts, and others). Malaria control/elimination strategies may be stratified by these divisions, and thus surveilling cases moving between and within second-level administrative units will help move countries toward sub-national elimination. While the WHO definition allows flexibility in the definition of the spatial unit defining an imported case, standardizing this definition may improve the organization’s ability to recognize and certify subnational elimination as incentivization for countries to continue toward total elimination.

In 175 articles, imported malaria was either explicitly defined or implicitly discussed as a case originating in an endemic country that was diagnosed in a non-endemic country, while 109 articles discussed imported cases between endemic countries. Articles that discussed imported malaria only between different countries predominantly focused on countries that were never malaria endemic or eliminated the disease long ago. Though few papers specifically discussed border (n = 7) and transnational (n = 3) malaria, the most discussed border area was between China and Myanmar.

Within-country mobility and its implications for imported malaria was far less frequently discussed; only 22 articles discussed imported malaria between an endemic and a non-endemic region in the same country, while 28 articles discussed imported malaria as a case travelling between two endemic regions in the same country. The most discussed countries for within-country importation were Brazil (n = 8), China (n = 6), and South Africa (n = 3). Outlining these designations are foundational components to imported malaria and specifically subnational malaria elimination goals, and they are the baseline that a minimum standard of data should determine for each reporting case. Beyond these core designations, further details reported in the literature included nationality of the infected individual, whether the sending and receiving locations were malaria endemic, parasite species, and parasite drug resistance.

Data sources used to identify imported malaria cases were diverse and varied in quality and by country. Most high-income countries leveraged government collected national malaria surveillance systems and case registries or hospital records with rich data extending beyond the minimum standard to designate imported status, which we describe below. In low- and middle-income countries, the predominant data source was either surveillance systems with lapses in coverage, or reliance on data collected specifically for a study. Two examples of upper-middle-income countries with strong national surveillance systems with mandatory reporting of all diagnosed cases are China and Brazil. Both countries, despite their geographical size, routinely collect detailed and high-quality data. In general, molecular diagnosis of malaria cases was rare (n = 10), all done for specific research, not on a programmatic and routine basis.

Only 11 articles discussed international importation of non-endemic parasites. The most cited species imported into a country was *Plasmodium ovale*. Of the 282 articles reviewed, 8.1% included a specific time between infection/travelling to the endemic region and time of notification in their definition of imported malaria, ranging from one week to 12 months. Countries with no endemic malaria transmission, except for those that have recently achieved elimination, commonly discussed imported malaria cases as any malaria case notifying within the country (n = 90). Other common metrics used to distinguish imported malaria cases from autochthonous cases were travel histories (n = 81), and epidemiological investigations (n = 29).

### Proposed conceptual framework and minimum data requirements

**Figure 2:**
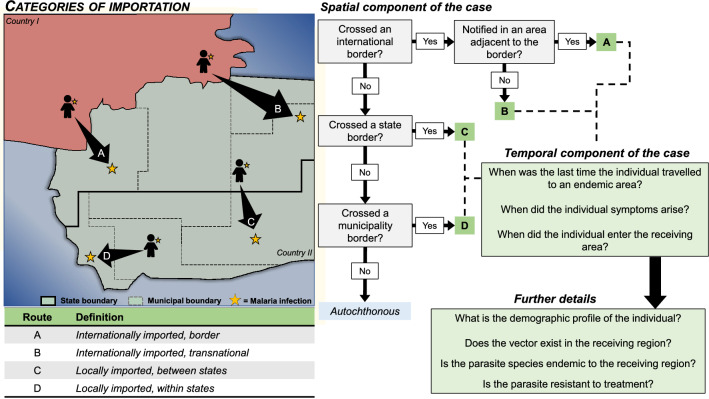
Data decision flow for determining categories of importation based on conceptual framework devised from scoping review. Minimum necessary data to define imported cases (i) date and location of notification, (ii) location of residence, and (iii) most likely location of infection. If (iii) is unavailable, (iv) date of travel, and (v) date of first symptom must be collected to deduce most likely location of infection.

Based on the scoping review, we proposed a conceptual framework for classifying imported malaria cases (Fig. 2), which extends WHO’s definition^15^ to include importation within a country. The framework groups the spatial component of the definition into four categories.

First, international border importation: a case travelling into a country from a bordering country and notifying in an administrative area along or within a specified distance from the international border^15^. Border malaria has challenged elimination in many countries and has contributed to the final cases existing in border administrative regions^31–36^. From the scoping review, studies on border malaria were mostly based on epidemiological surveillance data (71.4% of border malaria articles), and all were written from the perspective of the country nearer elimination.

Second, international transnational importation: cases travelling into a country from a malaria endemic country and notifying anywhere other than along or within a specified distance from an international border, including individuals travelling via land or sea^15^. These cases can originate from malaria endemic countries either bordering the receiving country or anywhere else globally and may or may not contribute to onward transmission in the receiving country. In malaria non-endemic countries, these cases are typically from individuals who travelled to a malaria-endemic country to visit friends or relatives (VFR travelers)^37–40^.

Third, locally imported, within administrative units: a case travelling between areas (localities, municipalities) within the same sub-national administrative unit (districts, states). An individual who was infected in one area, and who notified in another area within the same sub-national administrative unit, would be classified as locally imported within administrative units. These cases may over-burden specific health systems within an administrative unit, particularly in urban centers where travel networks are most connected and higher quality of care.

Fourth, locally imported, between administrative units: a case travelling between subnational administrative units. Individuals within the country may be travelling to more remote locations for economic opportunity, vacations, displacement, or other reasons^41–43^. Some countries may have administrative units that have achieved subnational malaria elimination but are still vulnerable to autochthonous transmission due to the existence of the malaria vector.

The third and fourth categories are particularly important for countries moving toward elimination or those who seek sub-national elimination of specific areas.

Based on the proposed framework, the minimum data countries seeking elimination need to routinely detect and classify imported cases are: (1) date and location of case notification, (2) location of residence, and (3) most likely location of infection (Fig. 2). Metrics for (1) and (2) are likely to be routinely collected if countries have surveillance systems that record malaria cases and basic demographics of patients. Metric (3) requires an epidemiological investigation (travel history in the past two weeks to a month) of cases in which the location of notification differs from the location of residence. In this case, not only imported cases can be detected and quantified, but also the specific information of sending and receiving locations would be available, faciliting network analysis of parasite flow.

Countries like Brazil have demonstrated the capability of routinely collecting these data across a vast network of laboratories. Next, we use the case of Brazil to demonstrate how a malaria surveillance system can apply the proposed framework to define imported malaria cases and the importance of tracking importation.

### Imported malaria case study: Brazil’s malaria epidemiological surveillance information system

Brazil meets the minimum data criteria for detecting and classifying imported malaria cases and allows for fine-scale spatiotemporal tracking of imported malaria. Classification of imported/autochthonous cases was possible considering infection and notification locations, and results of the epidemiological investigation that identified the likely place of infection. Cases were defined as autochthonous case if the notification municipality was the same as the infection municipality, regardless of residence location. An imported case was defined as a case in which the notification municipality was different from the infection municipality/country, regardless of residence location. Imported cases could be further broken down by cross-border (infection acquired abroad, notification was either border or transnational), or local importation (infection acquired in a different municipality than notification location, either between or within states).

**Figure 3:**
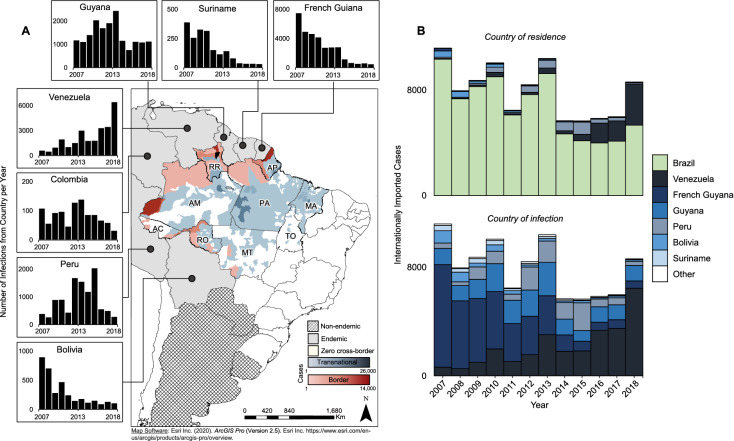
Descriptive plots of cross-border malaria burden in Brazil and case origins. (**A**) Map shows the intensity of transnational and border cases in Brazil between 2007 and 2018. State acronyms: AC = Acre, AP = Amapá, AM = Amazonas, PA = Pará, RO = Rondônia, RR = Roraima, TO = Tocantins, MA = Maranhão, MT = Mato Grosso. Bar charts demonstrate the number of cases annually entering Brazil from surrounding endemic countries. (**B**) Relative contribution of cases internationally imported into Brazil. Top bar chart details the country of residence; bottom bar chart details the country of infection. Maps created using ESRI ArcGIS Pro v2.5 (Esri Inc. (2020). *ArcGIS Pro* (Version 2.5). Esri Inc. https://www.esri.com/en-us/arcgis/products/arcgis-pro/overview.).

Between 2007 and 2018, Brazil reported 4,122,232 malaria cases (Supplementary Table S2). Of those, 15.41% could be classified as imported. The most common form of importation was between municipalities within the same state (70.32%), followed by between states (14.71%), transnational malaria (8.89%), and border malaria (6.08%). Bordering countries were responsible for > 99% of all cross-border cases, and the number of cases coming from each bordering country varied spatially and temporally. Between 2007 and 2013, cases imported from French Guiana were most common, and since then Venezuela has been the main source (Fig. 3A)^44^. Most internationally imported cases have been Brazilian residents infected in other countries, though in recent years Venezuelan residents migrating into Brazil has increased and became the majority (Fig. 3B).

**Figure 4:**
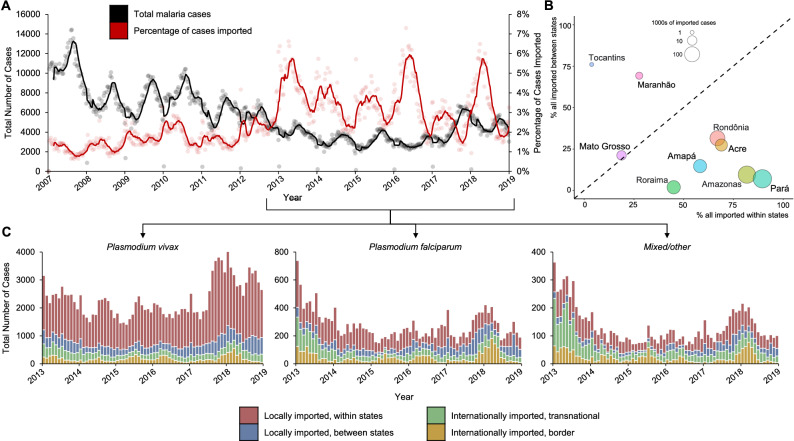
Dynamics of cases by spatial level and species. (**A**) Time series of total malaria cases (black) and percentage of those cases that were imported (red). Dots are daily number of cases, lines are 30-day moving averages of cases. (**B**) Bivariate plot of percentage of all imported cases occurring within and between states, with bubble size corresponding to the total number of imported cases in each state from 2007 to 2018. (**C**) Species and importation type breakdown of cases between 2013 and 2018. Bars are monthly increments.

Although the total number of cases were declining since 2009, beginning in 2013 the proportion of total cases that were imported increased (Fig. 4A). Between state importation was more prevalent in states where malaria transmission is low (Fig. 4B). Although not part of the proposed minimum data, we assessed the type of parasite and showed that between 2013 and 2018 the parasite species infecting individuals varied by importation type (Fig. 4C). *P. vivax* was the most common species of infection (84.97%) and was particularly prevalent in cases imported within the same state (84.70%). *P. falciparum* and mixed/other parasite species cases were proportionally more prevalent among cross-border cases than among within-country imported cases.

**Figure 5:**
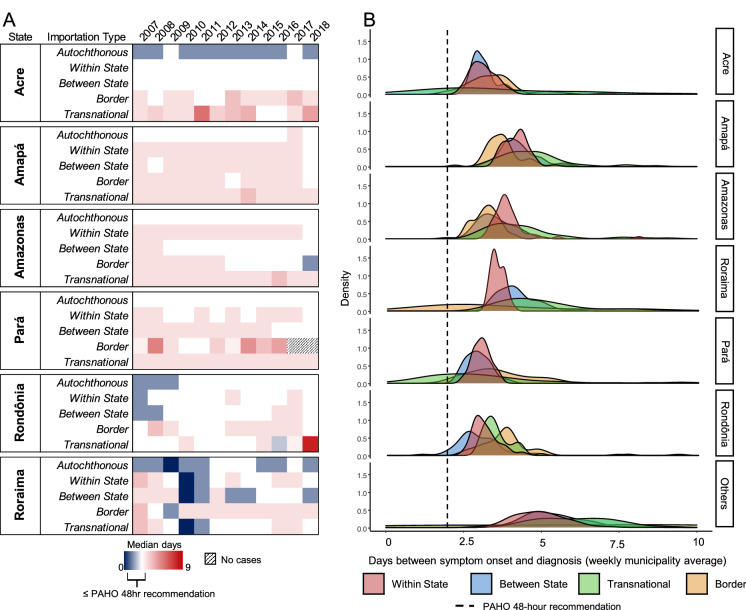
Analysis of time between symptom onset and diagnosis to assess Brazil’s health system response capabilities. (**A**) Heatmap demonstrating the median number of days that passed between symptom onset and malaria diagnosis compared to the PAHO 48 h recommendation. States are broken down into the different types of malaria cases denoted from the proposed conceptual framework. (**B**) Density plots of the distribution of days between symptom onset and diagnosis based on weekly municipality averages and split by case type. The dotted line represents PAHO’s 48 h recommendation.

We also calculated the time between the date of onset of symptoms and the date of diagnosis. In Brazil, this period varied by importation type and state (Fig. 5). An average of 2.71 days (SD = 7.05 days) occurred among autochthonous cases, 4.59 days (SD = 12.64 days) among transnational cases, 3.65 days (SD = 7.07 days) among border cases, 3.57 days (SD = 8.34 days) among within-state cases, and 3.68 days (SD = 8.45 days) among between-state cases. The distribution of time from symptom onset and diagnosis was very skewed for all states, and the mode of the distruibution was above the 48 h recommended by PAHO.

## Discussion

We conducted a scoping review on the definitions for imported malaria used between January 2010 and December 2020; 282 studies were scrutinized to determine the country/region of focus, the ways in which imported malaria was defined, and specific imported case details. The ability to detect and classify imported malaria vary dramatically posing challenges to in- and cross-country malaria control strategies. Based on our review, we revisited the definition for imported malaria, suggested the minimum data necessary to monitor imported malaria, and used publicly available surveillance data from Brazil as a case to demonstrate its use and importance.

Four types of imported cases are proposed: (1) international border, (2) international transnational, (3) within administrative units of a country, and (4) between administrative units of a country. Types (1) and (2) were the most commonly reported in our scoping review. The scarcity of studies focusing on importation within countries is worrisome in the context of countries near elimination where local transmission is often geographically restricted. First, mobility of infected individuals within country borders may spike transmission in receptive, vulnerable areas and cases may more frequently present as severe due to naïve immune systems of local individuals^45^. Second, mobility of infection individuals may result in the reintroduction of malaria in areas that have eliminated the disease, but that remain receptive, hampering elimination efforts. Types (3) and (4) were the most common types of importation in the Brazilian example, although currently not closely tracked by control efforts.

The WHO recommends that countries near elimination or with very low transmission classify cases as imported or autochthonous, but at higher levels of transmission this information should not be of primary focus^27^. Our findings are most applicable to those countries near elimination or with very low transmission. In these contexts, subnational malaria elimination is stated in the WHO-GTS to be a vital step on the path toward malaria elimination, and tracking within-country importation is key to sustaining levels of zero transmission in subnational areas that have eliminated the disease. In very-low transmission and elimination settings, assessing within-country importation in addition to between-country is thus paramount to meet the WHO-GTS for malaria^12^. Building and/or scaling up surveillance systems as a core intervention for malaria in line with the WHO-GTS is key in tracking and treating imported cases quickly in fragile elimination settings. While our findings and recommendations are less relevant for high burden countries, where the focus is on control, they will gain importance as those countries make strides in malaria control and move toward elimination.

Our scoping review demonstrated that international travel between endemic and non-endemic countries was the most discussed movement, with studies focused on non-endemic countries. The average 2018 GDP per capita (weighted by number of studies) of the malaria endemic countries represented in the literature review was 15,801USD, while the average 2018 GDP per capita of all malaria non-endemic countries was 45,724USD. Low-and-middle income countries may lack capacity to identify imported cases because national surveillance systems may be expensive and demand adequately equipped and organised systems to monitor and collect data. This, however, presents an opportunity for cross-country collaboration. High-income countries often use molecular techniques and travel history to determine the most likely location of infection of all notifying cases^46–54^. Sharing data and expertise with endemic countries where travelers became infected may aid low- and middle-income countries that lack capacity to collect their own data and is crucial if molecular techniques are used to determine drug-resistance status.

Global surveillance systems have been developed to this effect. For example, GeoSentinel, a global collaboration of 66 clinics monitoring infectious diseases among international travelers, “is based on the concept that these clinics are ideally situated to effectively detect geographic and temporal trends in morbidity among travelers, immigrants and refugees”^55^. This effort has made great strides in developing a publicly available data system for tracking imported infections, but the distribution of data collection sites is heavily skewed toward high-income nations (48 of the 66 are distributed across Europe and the USA). Though it may be financially or logistically unfeasible for low-income countries to adapt a system such as GeoSentinel, international efforts to expand access to this global database may be an interim solution while surveillance systems are ramping up. As for country specific registry of imported cases, Spain’s +Redivi is free to access via the internet and collects detailed epidemiological data on every malaria case notifying in the country^56,57^.

Throughout the process from control to elimination of malaria, regions of a specific country may remain both receptive to re-infection and vulnerable (i.e., importation risk) to infected individuals (with endemic malaria species, new species, or drug-resistant parasites). Regional elimination of malaria, which is possible while other areas of a country are experiencing high transmission rates, may create areas with no transmission that are at risk of importation and reestablishment of the disease^58^. Here, we argue that tracking the different levels of importation through the collection of minimum data is vital to using surveillance as a core intervention, and for sustaining regional elimination. Internationally imported cases may pose less of a threat to sub-national elimination (particularly for administrative areas not on the border) than within-country importation, and require different tactics for control. Control of internationally imported cases by definition focuses on tracking and treating those individuals, while controlling within-country importation requires a larger suite of tools to control transmission at the source of the infection, control further transmission in the location receiving the infection, and to track and treat mobile individuals that may follow common travel patterns. All forms of surveillance are important to achieve subnational and national elimination. Once malaria elimination is achieved, receptivity and vulnerability of regions should continue to be regularly reported to prevent the re-establishment of the disease at the country level. Greece offers a prime example of a setting that has achieved elimination but is receptive to malaria and uses active case detection to regularly track importation risk to prevent local transmission from occurring^59–61^.

The WHO has laid out a stepwise process of scaling up surveillance as an intervention for countries in different stages of malaria control and elimination^27^. Here we argue that surveillance systems at all levels of malaria control and elimination need to collect minimum data to detect and classify imported cases, and collection can be facilitated by a dense network of health posts and community health workers. Tracking cross-border and internal movement can dramatically improve malaria intervention stratification^44^.

Our suggestion of minimum data to detect and classify imported malaria cases raises issues of financial and human resources, particularly in low-income malaria endemic countries. In addition, it has some challenges. First, asymptomatic malaria infections are not captured by surveillance, as individuals without symptoms do not seek medical care, but can contribute to local transmission patterns. If the importation happens in endemic areas, the fact that an imported asymptomatic case may have led to secondary infections will be missed. If the importation happens in an area free of malaria, then secondary cases would trigger immediate responses to prevent further transmission. Second, foreigners may report their residence location incorrectly when they seek medical care in border areas, resulting in underestimation of cross-border cases. Similarly, individuals may misreport their residency location when seeking care locally, which would underestimate within-country importation. Despite these challenges, the Brazilian case presented here indicates that these minimum data provide much needed information to incorporate international and local importation of malaria in stratification efforts towards malaria elimination.

While the first step is to collect the minimum data needed to track imported malaria, the next step must be to use the data for decision making, and thus mitigate the potential consequences of importantion. Here we show that despite having the data, the time between symptoms onset and diagnosis in Brazil is still above the recommended period, which may contribute to a larger number of secondary cases.

In summary, the collection of minimum data could facilitate the monitoring of imported malaria, and the use of a common definition would help comparisons across countries. Both would aid in establishing stronger cross-country collaboration, sharing of data, and strengthening of surveillance systems to help achieve elimination goals outlined in the WHO GTS for malaria. The presented case for Brazil highlights the capabilities of regular surveillance systems to monitor importation, but also the need to regularly use these data for informing local responses. Specifically, results show the importance of in-country importation, a topic often neglected in surveillance and local policies.

## Supplementary Information

Supplementary Information.

## Data Availability

Aggregated data can be found on GitHub (https://github.com/mcastrolab/Imported_malaria_review).
