# Supplementary material for: Imported malaria definition and minimum data for surveillance
Source: Sci Rep. 2022 Oct 26;12:17982. doi: 10.1038/s41598-022-22590-6 (PMC9605982; doi:10.1038/s41598-022-22590-6)
Supplement: Supplementary file 1 — Supplementary Information. [file 41598_2022_22590_MOESM1_ESM.docx]

**Supplementary appendix**

**Imported Malaria Definition and Minimum Data for Surveillance**

Nicholas J. Arisco, MS^1^; Cassio Peterka, MS^2^; Marcia C. Castro, PhD^1*^

^1^ Department of Global Health and Population, Harvard T.H. Chan School of Public Health, Boston, MA, 02115, USA

^2^ Secretaria de Vigilância em Saúde, Ministério da Saúde, Brasília, DF, 70723-040, Brazil

*Corresponding author

**Table S1. Characteristics of the studies included in the review.**

| **Categories** | | **Number of Studies (%)** | | **Citations** |
| --- | --- | --- | --- | --- |
| Definition adopted | |  | |  |
|  | World Health Organization | 33 | (11.7%) | [S1-S33] |
|  | Technical Scheme China | 33 | (11.7%) | [S34-S66] |
|  | Other definition | 31 | (11.0%) | [S67-S97] |
|  | None explicitly listed | 185 | (65.6%) | [S98-S282] |
| Spatial scale |  |  |  |  |
|  | Within Country, Endemic to Non-Endemic | 22 | (7.8%) | [S6, S39, S46, S57, S68, S74, S83, S88, S90, S92, S98, S107, S121, S132, S141, S160, S183, S201, S245, S250, S257, S266] |
|  | Within Country, Endemic to Endemic | 28 | (9.9%) | [S14, S34, S39, S46, S72, S74, S76, S83, S88, S90, S92, S95, S98, S107, S121, S132, S183, S201, S212, S222, S248, S250, S257, S266, S270, S272, S276, S282] |
|  | Between Country, Endemic to Non-Endemic | 175 | (62.1%) | [S1-S5, S7-S13, S15-S17, S19-S23, S26-S28, S31, S32, S53, S55, S58, S62, S64, S67-S71, S73-S75, S77, S82, S84, S85, S96-S106, S108-S110, S112-S120, S122-S125, S127-S131, S133, S136-S140, S142, S144, S146-S148, S150-S159, S163, S164, S166, S167, S169-S172, S176, S178-S182, S184-S187, S189-S191, S194-S196, S199, S200, S202, S203, S206, S207, S209-S211, S213, S215-S220, S223-S230, S232, S234, S236-S238, S240, S241, S243-S247, S249, S251, S252, S254-S256, S258, S262, S263, S267-S269, S271, S273, S274, S277-S281] |
|  | Between Country, Endemic to Endemic | 109 | (38.7%) | [S6, S14, S17-S19, S24, S25, S29, S30, S33-S52, S54-S57, S59-S66, S72, S74, S78-S81, S83, S86, S87, S89-S95, S98, S107, S111, S126, S132, S134, S135, S141, S143, S145, S149, S150, S157, S160-S162, S165, S173-S175, S177, S179, S183, S188, S192, S193, S197, S198, S201, S204, S208, S212, S214, S221, S231, S233, S235, S239, S242, S250, S253, S260, S261, S264-S266, S270, S272, S275, S276, S280] |
|  | Explicit mention of “border” spatial scale | 7 | (2.5%) | [S46, S66, S93, S95, S214, S239, S250] |
|  | Explicit mention of “transnational” spatial scale | 3 | (1.1%) | [S93, S95, S250] |
| Data source |  |  |  |  |
|  | Government data | 130 | (46.1%) | [S3, S8, S14, S18, S23, S26, S28, S29, S32-S37, S39, S41-S48, S51-S56, S58-S69, S72, S73, S75, S76, S78, S80, S83-S87, S89-S93, S95, S96, S101, S105, S108, S113, S115, S116, S121, S124, S126, S128, S130, S132, S135, S137, S138, S141, S143, S147, S148, S153, S155, S158, S162, S163, S166, S171, S173, S175, S181-S183, S186, S188-S190, S201, S212, S215, S216, S218, S219, S221, S224-S226, S233, S234, S236, S237, S244-S246, S249, S250, S252, S259, S260, S262-S266, S268, S269, S271, S272, S274, S276, S278-S280] |
|  | Hospital/clinic records (non-Smolecular) | 76 | (27.0%) | [S2, S4, S5, S10, S11, S13, S16, S20-S22, S24, S27, S31, S38, S70, S71, S74, S77, S82, S94, S99, S100, S102-S104, S110, S112, S114, S117, S118, S120, S122, S125, S127, S139, S140, S142, S149, S152, S154, S159, S160, S167, S169, S170, S172, S177, S178, S180, S185, S187, S191, S194, S196-S199, S203, S209, S214, S217, S220, S223, S228-S230, S238, S241, S243, S247, S254-S257, S267, S273] |
|  | Hospital/clinic records (molecular) | 10 | (3.5%) | [S50, S111, S129, S131, S202, S204, S205, S242, S251, S275] |
|  | Empirical data | 11 | (3.9%) | [S81, S145, S161, S164, S176, S192, S208, S222, S261, S270, S282] |
|  | Other | 55 | (19.5%) | [S1, S6, S7, S9, S12, S15, S17, S19, S25, S30, S40, S49, S74, S79, S88, S97, S98, S106, S107, S109, S119, S123, S133, S134, S136, S144, S146, S150, S151, S156, S157, S165, S168, S174, S179, S184, S193, S195, S200, S206, S207, S210, S211, S213, S227, S231, S232, S235, S239, S240, S248, S253, S258, S277, S281] |

| Metric to define importation | |  |  |  |
| --- | --- | --- | --- | --- |
|  | All cases aSumed imported | 90 | (31.9%) | [S1, S2, S4, S5, S7, S10, S11, S15, S16, S20-S23, S27, S28, S31, S70, S97, S99, S101-S103, S105, S106, S108, S110, S112, S113, S115, S116, S118, S120, S127, S129-S131, S136, S137, S139, S140, S144, S147, S148, S151, S153, S158, S159, S163, S166, S167, S169-S171, S178, S181, S182, S185, S187, S189-S191, S194, S196, S204-S207, S209, S210, S213, S215, S217, S219, S220, S234, S236, S241, S243, S244, S246, S249, S254, S255, S259, S263, S267, S271, S273, S279, S281] |
|  | Travel history | 80 | (28.4%) | [S3, S14, S17, S29, S30, S32, S33, S36-S38, S40-S42, S45, S47, S49, S50, S52-S56, S58-S60, S64-S66, S68, S69, S71-S73, S75, S77, S78, S80-S87, S89-S93, S96, S104, S109, S117, S124, S128, S143, S145, S154, S155, S173, S174, S183, S186, S198, S203, S208, S212, S214, S222, S226, S228, S229, S231, S233, S238, S257, S260, S270, S274, S276] |
|  | Epidemiological investigation | 28 | (9.9%) | [S8, S24, S34, S35, S39, S43, S44, S62, S67, S76, S79, S94, S95, S126, S142, S172, S179, S188, S199, S201, S221, S237, S250, S256, S261, S266, S269, S272] |
|  | Cellphone data | 2 | (0.7%) | [S88, S121] |
|  | Other/none listed | 82 | (29.1%) | [S6, S9, S12, S13, S18, S19, S25, S26, S46, S48, S51, S57, S61, S63, S74, S98, S100, S107, S111, S114, S119, S122, S123, S125, S132-S135, S138, S141, S146, S149, S150, S152, S156, S157, S160-S162, S164, S165, S168, S175-S177, S180, S184, S192, S193, S195, S197, S200, S202, S211, S216, S218, S223-S225, S227, S230, S232, S235, S239, S240, S242, S245, S247, S248, S251-S253, S258, S262, S264, S265, S268, S275, S277, S278, S280, S282] |
| Country receiving cases | |  |  |  |
|  | China | 51 | (18.1%) | [S18, S32, S34-S54, S56-S66, S141, S165, S173, S175, S186, S192, S197, S198, S204, S208, S233, S242, S245, S258, S265, S268, S280] |
|  | United States | 18 | (6.4%) | [S2, S20, S71, S75, S108, S113, S119, S158, S163, S184, S196, S209, S216, S225, S229, S234, S252, S279] |
|  | Spain | 12 | (4.3%) | [S102, S104, S106, S109, S112, S122, S159, S178, S202, S207, S210, S271] |
|  | France | 13 | (4.6%) | [S21, S31, S97, S100, S170, S187, S203, S215, S247, S254, S273, S281] |
|  | Italy | 11 | (3.9%) | [S27, S69, S117, S151, S152, S205, S220, S241, S243, S255, S278] |
|  | Sri Lanka | 10 | (3.5%) | [S8, S145, S180, S195, S199, S213, S218, S223, S237, S269] |
|  | United Kingdom | 14 | (5.0%) | [S70, S77, S103, S114, S146, S167, S168, S181, S185, S194, S206, S230, S236, S263] |
|  | Brazil | 8 | (2.8%) | [S14, S76, S95, S160, S201, S250, S272, S276] |
|  | South Africa | 7 | (2.5%) | [S33, S68, S72, S81, S92, S126, S162] |
|  | Saudi Arabia | 6 | (2.1%) | [S13, S174, S183, S193, S239, S270] |
|  | Other high-Sincome countries | 45 | (16.0%) | [S5, S7, S10-S12, S16, S23, S25, S26, S28, S67, S82, S84, S86, S93, S101, S105, S110, S111, S118, S120, S124, S125, S129, S131, S137, S139, S140, S147, S149, S153-S155, S211, S217, S219, S224, S227, S228, S238, S244, S249, S251, S256, S267, S275] |
|  | Other low-S and middle-Sincome countries | 61 | (21.6%) | [S6, S24, S29, S30, S73, S78-S80, S83, S85, S87-S91, S94, S96, S99, S107, S115, S116, S121, S127, S130, S132, S134-S136, S138, S142-S144, S148, S161, S164, S166, S169, S172, S176, S177, S179, S188, S191, S212, S214, S221, S222, S226, S231, S232, S235, S246, S248, S253, S257, S260-S262, S264, S266, S282] |
|  | Global/Multi-Scountry | 25 | (8.9%) | [S1, S3, S4, S9, S15, S17, S19, S22, S55, S74, S98, S123, S128, S133, S150, S156, S157, S171, S182, S189, S190, S200, S240, S259, S277] |
| Species imported |  |  |  |  |
|  | *Plasmodium ovale* | 8 | (2.8%) | [S58, S149, S177, S197, S233, S242, S258, S268] |
|  | *Plasmodium knowlesi* | 2 | (0.7%) | [S228, S237] |
|  | *Plasmodium malariae* | 3 | (1.1%) | [S47, S63, S236] |

| Time period since travel | |  |  |  |
| --- | --- | --- | --- | --- |
|  | 1 week | 2 | (0.7%) | [S94, S114] |
|  | 2 weeks | 4 | (1.4%) | [S77, S143]^, S^[S89] |
|  | 1 month | 39 | (13.8%) | [S30, S34-S66, S78, S104, S129, S208, S276] |
|  | 3 months | 34 | (12.1%) | [S1-S33, S90] |
|  | 6 months | 2 | (0.7%) | [S109, S205] |
|  | 12 months | 1 | (0.4%) | [S70] |
|  | None listed | 200 | (70.9%) | [S2-S29, S31-S36, S38-S40, S43-S53, S55-S58, S60-S63, S69, S71-S76, S79-S88, S91-S93, S95-S97, S105-S108, S110-S113, S115-S128, S130-S142, S144-S204, S206, S207, S209-S275, S277-S282] |
| Year published |  |  |  |  |
|  | 2010 | 14 | (5.0%) | [S1, S2, S67-S69, S98-S106] |
|  | 2011 | 13 | (4.6%) | [S70, S107-S118] |
|  | 2012 | 21 | (7.4%) | [S3-S6, S71-S73, S119-S131] |
|  | 2013 | 26 | (9.2%) | [S7-S9, S34, S35, S74, S75, S132-S150] |
|  | 2014 | 31 | (11.0%) | [S10-S15, S36-S39, S76, S77, S151-S170] |
|  | 2015 | 18 | (6.4%) | [S16-S19, S40, S78, S171-S182] |
|  | 2016 | 31 | (11.0%) | [S20-S22, S41-S47, S79-S83, S183-S198] |
|  | 2017 | 35 | (12.4%) | [S23-S25, S48-S55, S84-S86, S199-S219] |
|  | 2018 | 34 | (12.1%) | [S26, S27, S56, S87-S90, S220-S245] |
|  | 2019 | 36 | (12.8%) | [S28-S32, S57-S60, S91, S92, S246-S271] |
|  | 2020 (Until 12/1/20) | 23 | (8.2%) | [S33, S61-S66, S93-S97, S272-S282] |

**Table S2. Annual number of malaria cases in Brazil by species and importation type, 2007-2018.**

| **Year** | **Parasite sp.** | **Total Cases** | **Autochthonous** | **Imported Cases** | | | |
| --- | --- | --- | --- | --- | --- | --- | --- |
|  |  |  |  | **International** | | **Within Brazil** | |
|  |  |  |  | **Border** | **Transnational** | **Between States** | **Within States** |
| 2007 | *P. vivax* | 522,480 | 440,245 | 3,507 | 3,679 | 11,806 | 63,243 |
|  | *P. falciparum* | 111,531 | 91,168 | 1,364 | 1,217 | 3,380 | 14,402 |
|  | Mixed/other | 34,522 | 27,185 | 568 | 855 | 1,153 | 4,761 |
|  |  |  |  |  |  |  |  |
| 2008 | *P. vivax* | 378,957 | 318,165 | 2,188 | 2,988 | 10,128 | 45,488 |
|  | *P. falciparum* | 55,076 | 45,082 | 803 | 886 | 2,108 | 6,197 |
|  | Mixed/other | 17,413 | 13,730 | 417 | 678 | 611 | 1,977 |
|  |  |  |  |  |  |  |  |
| 2009 | *P. vivax* | 369,227 | 309,946 | 1,867 | 3,562 | 7,945 | 45,907 |
|  | *P. falciparum* | 57,991 | 47,377 | 718 | 1,313 | 1,701 | 6,882 |
|  | Mixed/other | 16,990 | 13,235 | 402 | 874 | 515 | 1,964 |
|  |  |  |  |  |  |  |  |
| 2010 | *P. vivax* | 415,772 | 354,501 | 2,511 | 4,237 | 7,267 | 47,256 |
|  | *P. falciparum* | 56,565 | 47,005 | 762 | 1,261 | 1,235 | 6,302 |
|  | Mixed/other | 17,035 | 13,343 | 410 | 892 | 441 | 1,949 |
|  |  |  |  |  |  |  |  |
| 2011 | *P. vivax* | 324,445 | 278,216 | 1,358 | 2,758 | 6,071 | 36,042 |
|  | *P. falciparum* | 35,142 | 29,005 | 514 | 1,019 | 804 | 3,800 |
|  | Mixed/other | 12,514 | 9,986 | 210 | 616 | 332 | 1,370 |
|  |  |  |  |  |  |  |  |
| 2012 | *P. vivax* | 280,417 | 239,016 | 2,353 | 3,293 | 5,984 | 29,771 |
|  | *P. falciparum* | 34,634 | 28,115 | 696 | 1,060 | 945 | 3,818 |
|  | Mixed/other | 16,731 | 13,864 | 367 | 650 | 352 | 1,498 |
|  |  |  |  |  |  |  |  |
| 2013 | *P. vivax* | 198,471 | 168,951 | 2,281 | 4,298 | 4,725 | 18,216 |
|  | *P. falciparum* | 29,399 | 24,325 | 789 | 1,416 | 611 | 2,258 |
|  | Mixed/other | 15,062 | 12,156 | 476 | 1,141 | 239 | 1,050 |
|  |  |  |  |  |  |  |  |
| 2014 | *P. vivax* | 162,521 | 138,793 | 1,456 | 2,863 | 3,214 | 16,195 |
|  | *P. falciparum* | 21,313 | 18,162 | 287 | 520 | 531 | 1,813 |
|  | Mixed/other | 8,058 | 6,742 | 218 | 338 | 166 | 594 |
|  |  |  |  |  |  |  |  |
| 2015 | *P. vivax* | 169,074 | 147,026 | 2,313 | 2,061 | 2,967 | 14,707 |
|  | *P. falciparum* | 15,250 | 12,824 | 422 | 383 | 366 | 1,255 |
|  | Mixed/other | 7,029 | 5,978 | 258 | 232 | 117 | 444 |
|  |  |  |  |  |  |  |  |
| 2016 | *P. vivax* | 156,731 | 134,023 | 1,975 | 2,431 | 3,422 | 14,880 |
|  | *P. falciparum* | 14,436 | 11,602 | 444 | 538 | 631 | 1,221 |
|  | Mixed/other | 7,146 | 5,997 | 146 | 328 | 207 | 468 |
|  |  |  |  |  |  |  |  |
| 2017 | *P. vivax* | 244,862 | 212,753 | 1,942 | 2,584 | 4,973 | 22,610 |
|  | *P. falciparum* | 19,734 | 16,541 | 409 | 515 | 1,028 | 1,241 |
|  | Mixed/other | 10,246 | 8,591 | 177 | 361 | 400 | 717 |
|  |  |  |  |  |  |  |  |
| 2018 | *P. vivax* | 253,828 | 216,458 | 2,819 | 3,644 | 5,991 | 24,916 |
|  | *P. falciparum* | 18,900 | 15,576 | 840 | 603 | 822 | 1,059 |
|  | Mixed/other | 12,730 | 11,109 | 346 | 366 | 300 | 609 |

**REFERENCES**

S1. P. E. Neave J, C. O. H.,Behrens, R. H. A review of risk factors for imported malaria in the European African diaspora. Journal of Travel Medicine. 2010.

S2. S. Mathai B, E.,Slim, J.,Nalmas, S. Severe malaria in immigrant population: a retrospective review. J Immigr Minor Health. 2010.

S3. B. L.-V. Monge-Maillo R. Migration and malaria in europe. Mediterranean Journal of Hematology and Infectious Diseases. 2012.

S4. H. H. Askling B, F.,Burchard, G.,Castelli, F.,Chiodini, P. L.,Grobusch, M. P.,Lopez-Vélez, R.,Paul, M.,Petersen, E.,Popescu, C.,Ramharter, M.,Schlagenhauf, P. Management of imported malaria in Europe. Malar J. 2012.

S5. L. C. Santos A, C. F.,Xerinda, S. M.,Tavares, M.,Lucas, R.,Sarmento, A. C. Severe imported malaria in an intensive care unit: a review of 59 cases. Malar J. 2012.

S6. T. Yangzom G, C. S.,Namgay, R.,Galappaththy, G. N.,Thimasarn, K.,Gosling, R.,Murugasampillay, S.,Dev, V. Malaria control in Bhutan: case study of a country embarking on elimination. Malar J. 2012.

S7. C. S. Lee G, D. B.,Church, D.,Laupland, K. B.,Eckhardt, R.,Ross, T.,Chan, W.,Pillai, D. R. Population-based laboratory surveillance of imported malaria in metropolitan Calgary, 2000-2011. PLoS One. 2013.

S8. G. N. Galappaththy F, S. D.,Abeyasinghe, R. R. Imported malaria: a possible threat to the elimination of malaria from Sri Lanka? Trop Med Int Health. 2013.

S9. K. Vliegenthart-Jongbloed dMM, M.,van Wolfswinkel, M. E.,Koelewijn, R.,van Hellemond, J. J.,van Genderen, P. J. Severity of imported malaria: protective effect of taking malaria chemoprophylaxis. Malar J. 2013.

S10. A. B. Evans K, D.,Banerji, A.,Boggild, A.,Kain, K. C.,Abdelhaleem, M.,Morris, S. K. Imported pediatric malaria at the Hospital for Sick Children, Toronto, Canada: a 16 year review. BMC Pediatr. 2014.

S11. A. G. Fonseca D, S. S.,Baptista, J. L.,Torgal, J. Imported malaria in portugal 2000-2009: a role for hospital statistics for better estimates and surveillance. Malar Res Treat. 2014.

S12. E. J. Nilles A, M.,Mohtasham, M. A.,Saif, M.,Sulaiman, L.,Seliem, R. M.,Kotlyar, S.,Dziura, J. D.,Al-Najjar, F. J. Epidemiological and clinical characteristics of imported malaria in the United Arab Emirates. J Travel Med. 2014.

S13. I. R. Musa G, G. I.,Eltoum, A. O.,Adam, I. Imported malaria at Buraidah Central Hospital, Qassim, Saudi Arabia: A retrospective analysis. Travel Medicine and Infectious Disease. 2014.

S14. R. B. P. Miguel PCdA, H.Coura, J. R.Moza, P. G.Costa, A. D. P.Brasil, P.Suarez-Mutis, M. C. Malaria in the state of Rio de Janeiro, Brazil, an Atlantic Forest area: an assessment using the health surveillance service. Memorias Do Instituto Oswaldo Cruz. 2014.

S15. S. Odolini G, P.,Kain, K. C.,Smith, K.,Leder, K.,Jensenius, M.,Coyle, C. M.,Castelli, F.,Matteelli, A. Imported Plasmodium vivax malaria ex Pakistan. J Travel Med. 2014.

S16. A. E. McCarthy M, C.,Prematunge, C.,Geduld, J. Severe malaria in Canada, 2001-2013. Malar J. 2015.

S17. H. J. W. Sturrock R, K. W.,Wegbreit, J.,Ohrt, C.,Gosling, R. D. Tackling imported malaria: an elimination endgame. Am J Trop Med Hyg. 2015.

S18. J. Feng X, H.,Xia, Z.,Zhang, L.,Xiao, N. Analysis of Malaria Epidemiological Characteristics in the People's Republic of China, 2004-2013. Am J Trop Med Hyg. 2015.

S19. M. Velarde-Rodríguez VdB, R.,Fergus, C.,Casellas, A.,Sanz, S.,Cibulskis, R.,Ramsay, A. R.,Bissell, K.,Zachariah, R. Origin of malaria cases: a 7-year audit of global trends in indigenous and imported cases in relation to malaria elimination. Glob Health Action. 2015.

S20. A. E. Goldman-Yassen M, V. K.,Arguin, P. M.,Daily, J. P. Higher Rates of Misdiagnosis in Pediatric Patients Versus Adults Hospitalized With Imported Malaria. Pediatr Emerg Care. 2016.

S21. F. Bruneel T, F.,Mira, J. P.,Houze, S.,Gibot, S.,Huisse, M. G.,Megarbane, B.,Choquet, C.,Corne, P.,Peytel, E.,Villers, D.,Camus, C.,Bouchaud, O.,Caumes, E.,Girard, P. M.,Simon, F.,Kalloumeh, A.,Roy, C.,Durand, R.,Le Bras, J.,Matheron, S.,Wolff, M. Imported falciparum malaria in adults: host- and parasite-related factors associated with severity. The French prospective multicenter PALUREA cohort study. Intensive Care Med. 2016.

S22. M. Marks A, M.,Whitty, C. J. M.,Doherty, J. F. Geographical and temporal trends in imported infections from the tropics requiring inpatient care at the Hospital for Tropical Diseases, London - a 15 year study. Transactions of the Royal Society of Tropical Medicine and Hygiene. 2016.

S23. A. Kanayama A, Y.,Matsui, T.,Kaku, K.,Kinoshita, H.,Oishi, K. Epidemiology of Imported Malaria Cases in Japan, 2006-2014: A Sentinel Traveler Surveillance Approach. Am J Trop Med Hyg. 2017.

S24. B. Dembele Y, A. A. Controlling imported malaria cases in the United States of America. Math Biosci Eng. 2017.

S25. B. S. Simon FAM, S. K.Al-Abri, S.Ali, O. A. M.Bonnot, G.Bienvenu, A. L.Petersen, E.Picot, S. An outbreak of locally acquired Plasmodium vivax malaria among migrant workers in Oman. Parasite. 2017.

S26. M. A. Chehab B, M. O.,Al-Dahshan, A.,Selim, N. A.,Al-Romaihi, H. E.,Al-Thani, M.,Farag, E. A. Evaluation of the Completeness and Timeliness of National Malaria Surveillance System in Qatar, 2016. Cureus. 2018.

S27. P. Zanotti O, S.,Tomasoni, L. R.,Grecchi, C.,Caligaris, S.,Gulletta, M.,Matteelli, A.,Cappa, V.,Castelli, F. Imported malaria in northern Italy: epidemiology and clinical features observed over 18 years in the Teaching Hospital of Brescia. Journal of Travel Medicine. 2018.

S28. E. Rovira-Vallbona B, E.,Guetens, P.,Verschueren, J.,Rebolledo, J.,Nulens, E.,Van der Hilst, J.,Clerinx, J.,Van Esbroeck, M.,Rosanas-Urgell, A. Imported malaria and artemisinin-based combination therapy failure in travellers returning to Belgium: A retrospective study. Travel Med Infect Dis. 2019.

S29. H. Vatandoost R, A.,Saghafipour, A.,Nikpour, F.,Nejati, J. Malaria situation in Iran: 2002-2017. Malaria Journal. 2019.

S30. J. L. Smith G, P.,Rijal, K. R.,Maglior, A.,Hollis, S.,Andrade-Pacheco, R.,Das Thakur, G.,Adhikari, N.,Thapa Shrestha, U.,Banjara, M. R.,Lal, B. K.,Jacobson, J. O.,Bennett, A. Designing malaria surveillance strategies for mobile and migrant populations in Nepal: a mixed-methods study. Malar J. 2019.

S31. L. Pull L, J. M.,Beardmore, M.,Michel, J. F.,Buffet, P.,Bouchaud, O.,Siriez, J. Y. Artenimol-piperaquine in children with uncomplicated imported falciparum malaria: experience from a prospective cohort. Malar J. 2019.

S32. S. S. Zhang F, J.,Zhang, L.,Ren, X.,Geoffroy, E.,Manguin, S.,Frutos, R.,Zhou, S. S. Imported malaria cases in former endemic and non-malaria endemic areas in China: are there differences in case profile and time to response? Infect Dis Poverty. 2019.

S33. J. G. Raman LB, R.Tessema, S.Brooke, B.Maharaj, R.Munhenga, G.Tshikae, P.Lakan, V.Mwamba, T.Makowa, H.Sangweni, L.Mkhabela, M.Zondo, N.Mohulatsi, E.Nyawo, Z.Ngxongo, S.Msimang, S.Dagata, N.Greenhouse, B.Birkholtz, L. M.Shirreff, G.Graffy, R.Qwabe, B.Moonasar, D. High levels of imported asymptomatic malaria but limited local transmission in KwaZulu-Natal, a South African malaria-endemic province nearing malaria elimination. Malaria Journal. 2020.

S34. X. F. Zhao Z, J. N.,Dong, H. J.,Zhang, T.,Bian, G. L.,Sun, Y. W.,Yao, M. H.,Chen, K. J.,Xu, G. Z. Epidemiological characteristics of malaria in Ningbo City, China 2000-2011. Trop Biomed. 2013.

S35. Y. Li W, G.,Sun, D.,Meng, F.,Lin, S.,Hu, X.,Wang, S. A case of Plasmodium ovale wallikeri infection in a Chinese worker returning from West Africa. Korean J Parasitol. 2013.

S36. J. Feng X, Z. G.,Vong, S.,Yang, W. Z.,Zhou, S. S.,Xiao, N. Preparedness for malaria resurgence in China: case study on imported cases in 2000-2012. Adv Parasitol. 2014.

S37. J. Feng Y, H.,Feng, X. Y.,Zhang, L.,Li, M.,Xia, Z. G.,Xiao, N. Imported Malaria in China, 2012. Emerging Infectious Diseases. 2014.

S38. X. Zhou H, J. L.,Njuabe, M. T.,Li, S. G.,Chen, J. H.,Zhou, X. N. A molecular survey of febrile cases in malaria-endemic areas along China-Myanmar border in Yunnan province, People's Republic of China. Parasite. 2014.

S39. Y. Liu H, M. S.,Zhou, H.,Wang, W.,Cao, Y.,Gosling, R. D.,Cao, J.,Gao, Q. Malaria in overseas labourers returning to China: An analysis of imported malaria in Jiangsu Province, 2001-2011. Malaria Journal. 2014.

S40. Z. Li Y, Y.,Xiao, N.,Zhou, S.,Lin, K.,Wang, D.,Zhang, Q.,Jiang, W.,Li, M.,Feng, X.,Yu, J.,Ren, X.,Lai, S.,Sun, J.,Fang, Z.,Hu, W.,Clements, A. C.,Zhou, X.,Yu, H.,Yang, W. Malaria imported from Ghana by returning gold miners, China, 2013. Emerg Infect Dis. 2015.

S41. J. Feng X, Z.,Zhang, L.,Cheng, S.,Wang, R. Risk Assessment of Malaria Prevalence in Ludian, Yongshan, and Jinggu Counties, Yunnan Province, After 2014 Earthquake Disaster. Am J Trop Med Hyg. 2016.

S42. J. W. Xu L, H. The relationship of malaria between Chinese side and Myanmar's five special regions along China-Myanmar border: a linear regression analysis. Malar J. 2016.

S43. Q. Zhang S, J.,Zhang, Z.,Geng, Q.,Lai, S.,Hu, W.,Clements, A. C.,Li, Z. Risk assessment of malaria in land border regions of China in the context of malaria elimination. Malar J. 2016.

S44. S. J. Lai W, N. A.,Huang, Z. J.,Bosco, C.,Sun, J. L.,Bird, T.,Wesolowski, A.,Zhou, S.,Zhang, Q.,Zheng, C. J.,Li, Z. J.,Tatem, A. J.,Yu, H. J. Plasmodium falciparum malaria importation from Africa to China and its mortality: an analysis of driving factors. Scientific Reports. 2016.

S45. S. Zhou L, Z.,Cotter, C.,Zheng, C.,Zhang, Q.,Li, H.,Zhou, S.,Zhou, X.,Yu, H.,Yang, W. Trends of imported malaria in China 2010-2014: analysis of surveillance data. Malar J. 2016.

S46. T. Hu L, Y. B.,Zhang, S. S.,Xia, Z. G.,Zhou, S. S.,Yan, J.,Cao, J.,Feng, Z. C. Shrinking the malaria map in China: measuring the progress of the National Malaria Elimination Programme. Infect Dis Poverty. 2016.

S47. Y. Cao W, W.,Liu, Y.,Cotter, C.,Zhou, H.,Zhu, G.,Tang, J.,Tang, F.,Lu, F.,Xu, S.,Gu, Y.,Zhang, C.,Li, J.,Cao, J. The increasing importance of Plasmodium ovale and Plasmodium malariae in a malaria elimination setting: an observational study of imported cases in Jiangsu Province, China, 2011-2014. Malar J. 2016.

S48. B. Shi Z, J.,Qiu, H.,Yang, G. J.,Xia, S.,Zhou, X. N. Risk assessment of malaria transmission at the border area of China and Myanmar. Infect Dis Poverty. 2017.

S49. K. Lin W, H.,Jiang, W.,Li, J.,Zhang, W.,Wei, S.,Yang, Y.,Huang, Y.,Feng, X.,Tu, H.,Feng, J. Malaria in the Guangxi Zhuang Autonomous Region in China: A Twelve-Year Surveillance Data Study. Am J Trop Med Hyg. 2017.

S50. M. Li L, J.,Xia, Z.,Xiao, N.,Jiang, W.,Wen, Y. A combined strategy for screening a clustered mobile population returning from highly endemic areas for Plasmodium falciparum. J Infect Dev Ctries. 2017.

S51. Q. Huang H, L.,Liao, Q. B.,Xia, J.,Wang, Q. R.,Peng, H. J. Spatiotemporal Analysis of the Malaria Epidemic in Mainland China, 2004-2014. Am J Trop Med Hyg. 2017.

S52. Q. Q. Shi C, P.,Zhang, C. X.,Guo, X. X.,Liu, L. J.,Wang, H. F.,Kou, J. X.,Huang, X. D.,Wang, H. W.,Gong, M. Q. Epidemiological analysis of 133 malaria cases in Shanxian County, Shandong Province, China. Asian Pacific Journal of Tropical Medicine. 2017.

S53. S. Lai L, Z.,Wardrop, N. A.,Sun, J.,Head, M. G.,Huang, Z.,Zhou, S.,Yu, J.,Zhang, Z.,Zhou, S. S.,Xia, Z.,Wang, R.,Zheng, B.,Ruan, Y.,Zhang, L.,Zhou, X. N.,Tatem, A. J.,Yu, H. Malaria in China, 2011-2015: an observational study. Bull World Health Organ. 2017.

S54. X. Kong L, X.,Tu, H.,Xu, Y.,Niu, J.,Wang, Y.,Zhao, C.,Kou, J.,Feng, J. Malaria control and prevention towards elimination: data from an eleven-year surveillance in Shandong Province, China. Malar J. 2017.

S55. Y. Liu S, H. J. W.,Yang, H.,Gosling, R. D.,Cao, J. The challenge of imported malaria to eliminating countries. Lancet Infect Dis. 2017.

S56. X. Zhang Y, L.,Sun, J.,Pan, J.,Chen, H.,Zhang, L.,Ruan, W. Malaria in Southeastern China from 2012 to 2016: Analysis of Imported Cases. Am J Trop Med Hyg. 2018.

S57. M. F. S. Domínguez García CVU, A.Bartolomé Moreno, C.Melús Palazón, E.Magallón Botaya, R. Imported malaria cases: The connection with the European ex-colonies. Malaria Journal. 2019.

S58. R. Zhou L, S.,Zhao, Y.,Yang, C.,Liu, Y.,Qian, D.,Wang, H.,Lu, D.,Zhang, H. Characterization of Plasmodium ovale spp. imported from Africa to Henan Province, China. Sci Rep. 2019.

S59. S. J. Lai S, J. L.,Ruktanonchai, N. W.,Zhou, S.,Yu, J. X.,Routledge, I.,Wang, L. P.,Zheng, Y. M.,Tatem, A. J.,Li, Z. J. Changing epidemiology and challenges of malaria in China towards elimination. Malaria Journal. 2019.

S60. T. Z. Wang SSF, J.Oo, M. M.Chen, J.Yan, C. F.Zhang, Y.Tie, P. Monitoring and evaluation of intervals from onset of fever to diagnosis before 1-3-7 approach in malaria elimination: a retrospective study in Shanxi Province, China from 2013 to 2018. Malaria Journal. 2019.

S61. D. W. She ZL, Q.Lu, L.Huang, Y.Zhang, K.An, D.Wu, J. Polymorphisms of pfcrt, pfmdr1, and K13-propeller genes in imported falciparum malaria isolates from Africa in Guizhou province, China. BMC Infectious Diseases. 2020.

S62. J. Xia W, D.,Wu, K.,Zhu, H.,Sun, L.,Lin, W.,Li, K.,Zhang, J.,Wan, L.,Zhang, H.,Liu, S. Epidemiology of plasmodium falciparum malaria and risk factors for severe disease in Hubei Province, China. American Journal of Tropical Medicine and Hygiene. 2020.

S63. J. Xia W, D.,Sun, L.,Zhu, H.,Li, K.,Zhang, J.,Lin, W.,Wan, L.,Zhang, H.,Liu, S. Characteristics of imported Plasmodium ovale spp. and Plasmodium malariae in Hubei Province, China, 2014-2018. Malar J. 2020.

S64. T. Yu F, Y.,Kong, X.,Liu, X.,Yan, G.,Wang, Y. Epidemiological characteristics of imported malaria in Shandong Province, China, from 2012 to 2017. Scientific reports. 2020.

S65. X. Song W, Y.,Kong, X. L.,Wang, H. F.,Huang, X. D.,Liu, H. M.,Liu, L. J.,Guo, X. X.,Zhang, C. X.,Zhao, Y. Q.,Kou, J. X.,Wang, H. W.,Cheng, P.,Gong, M. Q. Toward the Elimination of Malaria in China: A Retrospective Analysis of Malaria-Endemic Characteristics and Prevention Effects in Yantai, Shandong Province, 1951 to 2017. Vector-Borne and Zoonotic Diseases. 2020.

S66. X. T. Zhao T, W.,Lawawirojwong, S.,Wei, C.,Tang, Y. R.,Zhou, Y. W.,Sun, X. D.,Sattabongkot, J.,Kaewkungwal, J. Spatiotemporal Trends of Malaria in Relation to Economic Development and Cross-Border Movement along the China-Myanmar Border in Yunnan Province. Korean Journal of Parasitology. 2020.

S67. G. K. M. Lee T, K. W.,Goh, K. T.,Wilder-Smith, A. Trends in Importation of Communicable Diseases into Singapore. Annals Academy of Medicine Singapore. 2010.

S68. I. B. Weber B, L.,Mnyaluza, J.,Matjila, M. J.,Barnes, K.,Blumberg, L. The burden of imported malaria in Gauteng Province. S Afr Med J. 2010.

S69. R. Romi B, D.,D'Amato, S.,Cenci, C.,Peragallo, M.,D'Ancona, F.,Pompa, M. G.,Majori, G. Incidence of malaria and risk factors in Italian travelers to malaria endemic countries. Travel Medicine and Infectious Disease. 2010.

S70. D. Shingadia L, S. UK treatment of malaria. Arch Dis Child Educ Pract Ed. 2011.

S71. K. R. Schwartz V, R. Imported Pediatric Malaria Presenting to an Urban Pediatric Emergency Department A Case Series. Pediatric Emergency Care. 2012.

S72. L. Ngomane dJ, C. Changes in malaria morbidity and mortality in Mpumalanga Province, South Africa (2001-2009): a retrospective study. Malar J. 2012.

S73. M. Subelj S, M. Imported malaria in Slovenia, 2001-2011. Central European Journal of Medicine. 2012.

S74. C. S. Gueye S, K. C.,Galappaththy, G. N. L.,Rundi, C.,Tobgay, T.,Sovannaroth, S.,Gao, Q.,Surya, A.,Thakur, G. D.,Baquilod, M.,Lee, W. J.,Bobogare, A.,Deniyage, S. L.,Satimai, W.,Taleo, G.,Hung, N. M.,Cotter, C.,Hsiang, M. S.,Vestergaard, L. S.,Gosling, R. D. Active case detection for malaria elimination: a survey among Asia Pacific countries. Malaria Journal. 2013.

S75. K. A. Cullen A, P. M. Malaria surveillance--United States, 2011. MMWR Surveill Summ. 2013.

S76. Dlamini SK. Diagnosis and treatment of imported and odyssean malaria. S Afr Med J. 2014.

S77. M. Marks G-W, A.,Doherty, J. F.,Singer, M.,Walker, D. Managing malaria in the intensive care unit. Br J Anaesth. 2014.

S78. O. Maillard L, T.,Olivier, S.,Achirafi, A.,Aubert, L.,Lepere, J. F.,Thiria, J.,Pages, F.,Filleul, L. Major decrease in malaria transmission on Mayotte Island. Malaria Journal. 2015.

S79. E. V. B. Eer GH, H. Decreased endemic malaria in suriname: Moving towards elimination. American Journal of Tropical Medicine and Hygiene. 2016.

S80. F. Norouzinejad R, A.,Norouzinejad, A.,Ghaffari, F. Epidemiological status of malaria in Iran, 2011-2014. Asian Pacific Journal of Tropical Medicine. 2016.

S81. J. Raman M, N.,Frean, J.,Brooke, B.,Blumberg, L.,Kruger, P.,Mabusa, A.,Raswiswi, E.,Shandukani, B.,Misani, E.,Groepe, M. A.,Moonasar, D. Reviewing South Africa's malaria elimination strategy (2012-2018): progress, challenges and priorities. Malar J. 2016.

S82. L. T. Roggelin DN, B.Addo, M. M.Tannich, E.Rothe, C. Sharp increase of imported Plasmodium vivax malaria seen in migrants from Eritrea in Hamburg, Germany. Malaria Journal. 2016.

S83. S. Chihanga H, U.,Chanda, E.,Mosweunyane, T.,Moakofhi, K.,Jibril, H. B.,Motlaleng, M.,Zhang, W. Y.,Glass, G. E. Malaria elimination in Botswana, 2012-2014: achievements and challenges. Parasites & Vectors. 2016.

S84. E. Gilles S, D. P.,Anne, M.,Francois, C.,Nicole, G. Malaria cases in Switzerland from 2005 to 2015 and recent rise of imported Plasmodium vivax malaria. Swiss Medical Weekly. 2017.

S85. X. Dong Y, J.,Lou, L.,Zhu, L.,Feng, X.,Yao, L. Once malaria is eliminated, more attention should be paid to imported malaria: Data from five years of surveillance in the City of Yiwu in eastern China. Biosci Trends. 2017.

S86. Y. J. Choe C, S. A.,Cho, S. I. Importation of travel-related infectious diseases is increasing in South Korea: An analysis of salmonellosis, shigellosis, malaria, and dengue surveillance data. Travel Medicine and Infectious Disease. 2017.

S87. A. M. M. Kyaw K, S.,Das, M.,Thapa, B.,Linn, N. Y. Y.,Maung, T. M.,Lin, Z.,Thi, A. Alert-Audit-Act: assessment of surveillance and response strategy for malaria elimination in three low-endemic settings of Myanmar in 2016. Trop Med Health. 2018.

S88. F. A. H. Ihantamalala VR, F. M. J.Rakotondramanga, J. M.Cauchemez, S.Rahoilijaona, B.Pennober, G.Buckee, C. O.Rogier, C.Metcalf, C. J. E.Wesolowski, A. Estimating sources and sinks of malaria parasites in Madagascar. Nature Communications. 2018.

S89. M. H. Nghipumbwa A, S.,Kizito, W.,Takarinda, K. C.,Uusiku, P.,Mumbegegwi, D. R. Moving towards malaria elimination: trends and attributes of cases in Kavango region, Namibia, 2010-2014. Public Health Action. 2018.

S90. M. S. Lowa LS, M.Musonda, P. Human mobility and factors associated with malaria importation in Lusaka district, Zambia: a descriptive cross sectional study. Malaria Journal. 2018.

S91. A. J. DePina A, A. J. B.,Dia, A. K.,Moreira, A. L.,Furtado, U. D.,Baptista, H.,Faye, O.,Seck, I.,Niang, E. H. A. Spatiotemporal characterisation and risk factor analysis of malaria outbreak in Cabo Verde in 2017. Trop Med Health. 2019.

S92. R. Maharaj S, I.,Qwabe, B.,Mkhabela, M.,Kissoon, S.,Lakan, V. Decadal epidemiology of malaria in KwaZulu-Natal, a province in South Africa targeting elimination. Malar J. 2019.

S93. L. Hurtado C, A.,Rigg, C.,Perea, M.,Santamaria, A. M.,Chaves, L. F.,Moreno, D.,Romero, L.,Lasso, J.,Caceres, L.,Saldana, A.,Calzada, J. E. Long-term transmission patterns and public health policies leading to malaria elimination in Panama. Malaria Journal. 2020.

S94. M. N. Chipoya S-M, N. M. Prevalence, characteristics and risk factors of imported and local malaria cases in North-Western Province, Zambia: a cross-sectional study. Malar J. 2020.

S95. M. S. M. Gomes M, R. A. D.,Vieire, J. L. F.,Mendes, A. M.,Silva, G. D.,Peiter, P. C.,Suarez-Mutisf, M. C.,Franco, V. D.,Couto, Aard,Machado, R. L. D. Malaria in the borders between Brazil and French Guiana: social and environmental health determinants and their influence on the permanence of the disease. Saude E Sociedade. 2020.

S96. M. Tseroni G, M.,Baka, A.,Pinaka, O.,Pervanidou, D.,Tsironi, M.,Bleta, P.,Charvalakou, M.,Psinaki, I.,Dionysopoulou, M.,Legaki, A.,Vakali, A.,Patsoula, E.,Vassalou, E.,Bellou, S.,Diamantopoulos, V.,Georgakopoulou, T.,Mouchtouri, V.,Tsiodras, S.,Middleton, N.,Charalambous, A.,Raftopoulos, V.,Hadjichristodoulou, C. The Importance of an Active Case Detection (ACD) Programme for Malaria among Migrants from Malaria Endemic Countries: The Greek Experience in a Receptive and Vulnerable Area. International Journal of Environmental Research and Public Health. 2020.

S97. O. Bouchaud B, F.,Caumes, E.,Houzé, S.,Imbert, P.,Pradines, B.,Rapp, C.,Strady, C. Management and prevention of imported malaria. 2018 update of the 2007 French clinical guidelines. Med Mal Infect. 2020.

S98. B. Moonen C, J. M.,Snow, R. W.,Slutsker, L.,Drakeley, C.,Smith, D. L.,Abeyasinghe, R. R.,Rodriguez, M. H.,Maharaj, R.,Tanner, M.,Targett, G. Operational strategies to achieve and maintain malaria elimination. Lancet. 2010.

S99. E. D'Ortenzio S, D.,Dehecq, J. S.,Renault, P.,Filleul, L. Malaria imported into Réunion Island: is there a risk of re-emergence of the disease? Transactions of the Royal Society of Tropical Medicine and Hygiene. 2010.

S100. F. Bruneel T, F.,Corne, P.,Megarbane, B.,Mira, J. P.,Peytel, E.,Camus, C.,Schortgen, F.,Azoulay, E.,Cohen, Y.,Georges, H.,Meybeck, A.,Hyvernat, H.,Trouillet, J. L.,Frenoy, E.,Nicolet, L.,Roy, C.,Durand, R.,Le Bras, J.,Wolff, M. Severe imported falciparum malaria: a cohort study in 400 critically ill adults. PLoS One. 2010.

S101. G. G. Van Rijckevorsel S, G. J.,Geskus, R. B.,Wetsteyn, J. C.,Ligthelm, R. J.,Visser, L. G.,Keuter, M.,Van Genderen, P. J.,Van Den Hoek, A. Declining incidence of imported malaria in the Netherlands, 2000-2007. Malaria Journal. 2010.

S102. J. Arnáez R, M. A.,Albert, L.,Cogollos, R.,Rubio, J. M.,Villares, R.,Alarabe, A.,Cervera, A.,López-Vélez, R. Imported malaria in children: a comparative study between recent immigrants and immigrant travelers (VFRs). J Travel Med. 2010.

S103. M. Garbash R, J.,Whitty, C. J.,Chiodini, P. L.,Riordan, F. A.,Shingadia, D.,Ladhani, S. Intensive care admissions for children with imported malaria in the United kingdom. Pediatr Infect Dis J. 2010.

S104. P. Z. Fuertes P-A, A.,Molina, J. A. P.,Norman, F. F.,Monge-Maillo, B.,Navarro, M.,Lopez-Velez, R. Clinical and Epidemiological Characteristics of Imported Infectious Diseases in Spanish Travelers. Journal of Travel Medicine. 2010.

S105. S. Guedes Sa, H.,Kantele, A.,Lyytikainen, O. Imported malaria in Finland 1995 to 2008: An overview of surveillance, travel trends, and antimalarial drug sales. Journal of Travel Medicine. 2010.

S106. S. Rey Z, I.,Martinez-Mondejar, B.,Rubio, J. M.,Merino, F. J. Imported malaria in an area in southern Madrid, 2005-2008. Malaria Journal. 2010.

S107. A. Le Menach T, A. J.,Cohen, J. M.,Hay, S. I.,Randell, H.,Patil, A. P.,Smith, D. L. Travel risk, malaria importation and malaria transmission in Zanzibar. Sci Rep. 2011.

S108. C. R. Phares K, B. K.,Doney, A. C.,Arguin, P. M.,Green, M.,Mekonnen, L.,Galev, A.,Weinberg, M.,Stauffer, W. M. Presumptive treatment to reduce imported malaria among refugees from east Africa resettling in the United States. Am J Trop Med Hyg. 2011.

S109. E. Espinosa-Vega M-S, A. M.,Elcuaz-Romano, R.,Hernández-Febles, M.,Molina-Cabrillana, J.,Pérez-Arellano, J. L. Malaria in paradise: characterization of imported cases in Gran Canaria Island (1993-2006). J Travel Med. 2011.

S110. H. W. Unger M, A. D.,Ukachukwu, V.,McGoldrick, C.,Perrow, K.,Latin, G.,Norrie, G.,Morris, S.,Smith, C. C.,Jones, M. E. Imported malaria in Scotland--an overview of surveillance, reporting and trends. Travel Med Infect Dis. 2011.

S111. K. M. Choi C, Y. K.,Kang, Y. A.,Seo, S. Y.,Lee, H. W.,Cho, S. H.,Lee, W. J.,Rhie, H. G.,Lee, H. S.,Kim, J. Y. Study of the genetic discrimination between imported and autochthonous cases of malaria in South Korea. Journal of Travel Medicine. 2011.

S112. M. Garcia-Villarrubia M, J. P.,de Olalla, P. G.,Gascón, J.,Fumadó, V.,i Prat, J. G.,Treviño, B.,Pinazo, M. J.,Cabezos, J.,Muñoz, J.,Zarzuela, F.,Caylà, J. A. Epidemiology of imported malaria among children and young adults in Barcelona (1990-2008). Malar J. 2011.

S113. P. W. Hickey C, K. E.,Masuoka, P.,Campos, J. M.,Pastor, W.,Wong, E. C.,Singh, N.,Al-Rumhi, A.,Al-Hashami, Z.,Al-Hamidhi, S.,Gadalla, A.,Naeem, R.,Ranford-Cartwright, L.,Pain, A.,Sultan, A. A.,Babiker, H. A. A local, regional, and national assessment of pediatric malaria in the United States. J Travel Med. 2011.

S114. R. Bhome B, R. PCR negative cerebral malaria in a traveller returning from Mumbai. BMJ Case Rep. 2011.

S115. R. Neghina N, A. M.,Marincu, I.,Iacobiciu, I. International travel increase and malaria importation in Romania, 2008-2009. Vector Borne Zoonotic Dis. 2011.

S116. R. Neghina N, A. M.,Marincu, I.,Iacobiciu, I. Malaria and the Campaigns Toward its Eradication in Romania, 1923-1963. Vector-Borne and Zoonotic Diseases. 2011.

S117. S. Antinori C, B.,Galimberti, L.,Orlando, G.,Schifanella, L.,Milazzo, L.,Viola, A.,Giuliani, G.,Ridolfo, A.,Corbellino, M. Diagnosis and therapy for hospitalized imported malaria in adults in Italy. J Travel Med. 2011.

S118. Z. Daki? P, M.,Djurkovi?-Djakovi?, O.,Lavadinovi?, L.,Nikoli?, A.,Stevanovi?, G.,Poluga, J.,Ofori-Beli?, I.,Miloševi?, B.,Pavlovi?, M. Imported malaria in Belgrade, Serbia, between 2001 and 2009. Wien Klin Wochenschr. 2011.

S119. A. Agarwal M, M.,Arguin, P. M. The increase of imported malaria acquired in Haiti among US travelers in 2010. Am J Trop Med Hyg. 2012.

S120. A. E. Camburn I, R. J.,Holland, D.,Read, K.,Taylor, S. Imported malaria in Auckland, New Zealand. N Z Med J. 2012.

S121. A. Wesolowski E, N.,Tatem, A. J.,Smith, D. L.,Noor, A. M.,Snow, R. W.,Buckee, C. O. Quantifying the Impact of Human Mobility on Malaria. Science. 2012.

S122. B. C. Jiménez C-T, P.,Ruiz-Giardin, J. M.,Rojo-Marcos, G.,Cuadros-González, J.,Canalejo, E.,Cabello, N.,San Martín, J. V.,Barrios, A. M.,Hinojosa, J.,Molina, L. Imported malaria in pregnancy in Madrid. Malar J. 2012.

S123. E. J. Nilles A, P. M. Imported malaria: an update. Am J Emerg Med. 2012.

S124. K. Stark S, I. Increase in malaria cases imported from Pakistan to Germany in 2012. Eurosurveillance. 2012.

S125. R. Eckhardt B-F, L.,Ross, N. A.,Pillai, D. R.,Buckeridge, D. L. A spatial analysis of individual- and neighborhood-level determinants of malaria incidence in adults, Ontario, Canada. Emerg Infect Dis. 2012.

S126. R. Maharaj R, J. The feasibility of malaria elimination in south africa. American Journal of Tropical Medicine and Hygiene. 2012.

S127. R. Neghina N, E. D.,Nita, C.,Musta, V.,Nicoara, E.,Olariu, T. R. Two cases of imported malaria in Western Romania, 2010-2011. Asian Pacific Journal of Tropical Medicine. 2012.

S128. S. Odolini G, P.,Parola, P. Epidemiology of imported malaria in the mediterranean region. Mediterr J Hematol Infect Dis. 2012.

S129. T. J. Gray T, J. M.,Fairley, M.,Krause, V. L.,Markey, P. G. Imported malaria in the Northern Territory, Australia--428 consecutive cases. Commun Dis Intell Q Rep. 2012.

S130. V. Svihrova S, M.,Novakova, E.,Svihra, J.,Hudeckova, H. Costs analysis of the treatment of imported malaria. Malar J. 2012.

S131. Y. K. Mizuno YK, S.Takasaki, T. Imported malaria and dengue fever in returned travelers in Japan from 2005 to 2010. Travel Medicine and Infectious Disease. 2012.

S132. A. Fattahi Bafghi P, S. A.,Shamsi, F. Five-Year Status of Malaria (a Disease Causing Anemia) in Yazd, 2008-2012. Iran J Ped Hematol Oncol. 2013.

S133. C. J. M. Whitty C, P. L.,Lalloo, D. G. Investigation and treatment of imported malaria in non-endemic countries. Bmj-British Medical Journal. 2013.

S134. C. Simon M, K.,Mosweunyane, T.,Jibril, H. B.,Nkomo, B.,Motlaleng, M.,Ntebela, D. S.,Chanda, E.,Haque, U. Malaria control in Botswana, 2008-2012: the path towards elimination. Malar J. 2013.

S135. D. L. C. Smith JMC, C.Johnston, G.Gething, P. W.Gosling, R.Buckee, C. O.Laxminarayan, R.Hay, S. I.Tatem, A. J. A sticky situation: the unexpected stability of malaria elimination. Philosophical transactions of the Royal Society of London Series B, Biological sciences. 2013.

S136. F. Arslan M, A.,Batirel, A.,Inan, A.,Balkan,, II,Nazlican, O.,Uzun, C.,Vahaboglu, H. Imported Plasmodium falciparum malaria in Istanbul, Turkey: risk factors for severe course and mortality. Tropical Doctor. 2013.

S137. F. Higa T, M.,Tasato, D.,Karimata, Y.,Nakamura, H.,Miyagi, K.,Haranaga, S.,Hirata, T.,Hokama, A.,Cash, H. L.,Toma, H.,Fujita, J. Imported malaria cases in Okinawa prefecture, Japan. Japanese Journal of Infectious Diseases. 2013.

S138. H. M. Jamain AS, Q. A.,Kanani, K. A.,Askling, H. H.,Bruneel, F.,Burchard, G.,Castelli, F.,Chiodini, P. L.,Grobusch, M. P.,Lopez-Vélez, R.,Paul, M.,Petersen, E.,Popescu, C.,Ramharter, M.,Schlagenhauf, P. Epidemiological pattern of imported malaria in Jordan from 2007 to 2011. Trop Biomed. 2013.

S139. H. Siikamaki K, P.,Lyytikainen, O.,Kantele, A. Imported malaria in Finland 2003-2011: prospective nationwide data with rechecked background information. Malaria Journal. 2013.

S140. J. C. Yombi J, S.,Colin, G.,Van Gompel, F.,Bigare, E.,Belkhir, L.,Vandercam, B. Imported malaria in a tertiary hospital in Belgium: epidemiological and clinical analysis. Acta Clin Belg. 2013.

S141. J. H. Yin Y, M. N.,Zhou, S. S.,Wang, Y.,Feng, J.,Xia, Z. G. Changing malaria transmission and implications in China towards National Malaria Elimination Programme between 2010 and 2012. PLoS One. 2013.

S142. K. A. Braima S, J. S.,Ghazali, A. R.,Muslimin, M.,Jeffery, J.,Lee, W. C.,Shaker, M. R.,Elamin, A. E.,Jamaiah, I.,Lau, Y. L.,Rohela, M.,Kamarulzaman, A.,Sitam, F.,Mohd-Noh, R.,Abdul-Aziz, N. M. Is there a risk of suburban transmission of malaria in Selangor, Malaysia? PLoS One. 2013.

S143. K. Koita N, J.,Kunene, S.,Zulu, Z.,Ntshalintshali, N.,Gandhi, M.,Gosling, R. Targeting imported malaria through social networks: a potential strategy for malaria elimination in Swaziland. Malar J. 2013.

S144. K. Shkurti V, G.,Velo, E.,Boçari, A.,Kokici, M.,Kraja, D. Imported malaria in Albania and the risk factors that could allow its reappearance. Malar J. 2013.

S145. K. Wickramage P, R. G.,Peiris, S. L.,Mosca, D. High attack rate for malaria through irregular migration routes to a country on verge of elimination. Malaria Journal. 2013.

S146. M. L. Willcox M, J.,O'Dempsey, T. Imported malaria. Bmj. 2013.

S147. M. P. R. Nelder CW, D.Johnson, K.Li, L.Baker, S. L.Marshall, S.Bhanich-Supapol, W.Pillai, D. R.Ralevski, F. Spatiotemporal Dynamics and Demographic Profiles of Imported Plasmodium falciparum and Plasmodium vivax Infections in Ontario, Canada (1990-2009). PLoS ONE. 2013.

S148. Stepie? M. Malaria in Poland in 2011. Przegla?d epidemiologiczny. 2013.

S149. Y. Kang Y, J. A case of Plasmodium ovale malaria imported from West Africa. Korean J Parasitol. 2013.

S150. Z. Huang T, A. J. Global malaria connectivity through air travel. Malaria Journal. 2013.

S151. A. Casuccio DA, C.,Casuccio, N.,Di Carlo, P.,Immordino, P.,de Pina-Costa, A.,Brasil, P.,Di Santi, S. M.,de Araujo, M. P.,Suárez-Mutis, M. C.,Santelli, A. C.,Oliveira-Ferreira, J.,Lourenço-de-Oliveira, R.,Daniel-Ribeiro, C. T. Visiting Friends and Relatives (VFRs) travelers and imported malaria in the Palermo district (Sicily)-//-Malaria in Brazil: what happens outside the Amazonian endemic region. Ann Ist Super Sanita. 2014.

S152. A. de Pina-Costa B, P.,Di Santi, S. M.,de Araujo, M. P.,Suárez-Mutis, M. C.,Santelli, A. C.,Oliveira-Ferreira, J.,Lourenço-de-Oliveira, R.,Daniel-Ribeiro, C. T. Malaria in Brazil: what happens outside the Amazonian endemic region. Memórias do Instituto Oswaldo Cruz. 2014.

S153. A. G. Fonseca D, S. S.,Baptista, J. L.,Torgal, J. The burden of imported malaria in Portugal 2003 to 2012. J Travel Med. 2014.

S154. C. H. Møller D, K. Imported malaria is stable from Africa but declining from Asia. Dan Med J. 2014.

S155. C. Lau W, P.,Slaney, D. The importance of surveillance for informing pretravel medical advice: imported malaria in New Zealand 1997-2009. Vector Borne Zoonotic Dis. 2014.

S156. C. Ohrt R, K. W.,Sturrock, H. J.,Wegbreit, J. A.,Lee, B.,Gosling, R. Surveillance systems to facilitate malaria elimination. American Journal of Tropical Medicine and Hygiene. 2014.

S157. C. Smith Gueye G, M.,Newby, G.,Lourenco, C.,Uusiku, P.,Liu, J. Namibia's path toward malaria elimination: a case study of malaria strategies and costs along the northern border. BMC Public Health. 2014.

S158. D. Chia M, J. O.,Aronin, S. I.,Suarez, R.,Virata, M. D.,Igwe, C. A.,Quentzel, H.,Sadigh, M. A depiction of imported malaria in Connecticut. IDCases. 2014.

S159. G. Rojo-Marcos R-M, J. M.,Ramírez-Olivencia, G.,García-Bujalance, S.,Elcuaz-Romano, R.,Díaz-Menéndez, M.,Calderón, M.,García-Bermejo, I.,Ruiz-Giardín, J. M.,Merino-Fernández, F. J.,Torrús-Tendero, D.,Delgado-Iribarren, A.,Ribell-Bachs, M.,Arévalo-Serrano, J.,Cuadros-González, J. Comparison of imported Plasmodium ovale curtisi and P. ovale wallikeri infections among patients in Spain, 2005-2011. Emerg Infect Dis. 2014.

S160. J. C. Dos-Santos A, R. N.,Castiñeiras, C. M.,Lopes, S. C.,Albrecht, L.,Garcia, M. T.,Levy, C. E.,Moretti, M. L.,Lacerda, M. V.,Costa, F. T. Imported malaria in a non-endemic area: the experience of the university of Campinas hospital in the Brazilian Southeast. Malar J. 2014.

S161. J. C. Patel T, S. M.,Juliao, P. C.,Parobek, C. M.,Janko, M.,Gonzalez, L. D.,Ortiz, L.,Padilla, N.,Tshefu, A. K.,Emch, M.,Udhayakumar, V.,Lindblade, K.,Meshnick, S. R. Genetic Evidence of Importation of Drug-Resistant Plasmodium falciparum to Guatemala from the Democratic Republic of the Congo. Emerg Infect Dis. 2014.

S162. J. F. Opie RdP, L. A. The burden of imported malaria in Cape Town, South Africa. South African Medical Journal. 2014.

S163. K. A. Cullen A, P. M. Malaria surveillance--United States, 2012. MMWR Surveill Summ. 2014.

S164. K. Kanani A, Z. S.,Shadfan, B.,Al-Rashadan, M.,Bani Hani, R. A retrospective study on imported malaria in Jordan. 1. Malaria among Jordanian UN peacekeeping forces. Bull Soc Pathol Exot. 2014.

S165. M. Dhimal A, B.,Kuch, U. Malaria control in Nepal 1963-2012: challenges on the path towards elimination. Malar J. 2014.

S166. M. St?pie? Rs, M. Imported malaria in Poland 2003 to 2011: implications of different travel patterns. J Travel Med. 2014.

S167. P. E. Neave B, R. H.,Jones, C. O. You're losing your Ghanaianess: understanding malaria decision-making among Africans visiting friends and relatives in the UK. Malar J. 2014.

S168. P. E. Neave J, C. O.,Behrens, R. H. Challenges facing providers of imported malaria-related healthcare services for Africans visiting friends and relatives (VFRs). Malar J. 2014.

S169. S. C. Chen C, H. L.,Chen, K. T. The epidemiology of imported malaria in Taiwan between 2002-2013: the importance of sensitive surveillance and implications for pre-travel medical advice. Int J Environ Res Public Health. 2014.

S170. T. Pistone D, A.,Mechain, M.,Receveur, M. C.,Malvy, D. Epidemiology of imported malaria give support to the hypothesis of 'long-term' semi-immunity to malaria in sub-Saharan African migrants living in France. Travel Med Infect Dis. 2014.

S171. A. K. Käser A, P. M.,Chiodini, P. L.,Smith, V.,Delmont, J.,Jiménez, B. C.,Färnert, A.,Kimura, M.,Ramharter, M.,Grobusch, M. P.,Schlagenhauf, P. Imported malaria in pregnant women: A retrospective pooled analysis. Travel Med Infect Dis. 2015.

S172. A. Kuna G, M.,Szostakowska, B.,Nahorski, W. L.,Myjak, P.,Stanczak, J. Imported Malaria in the Material of the Institute of Maritime and Tropical Medicine: A Review of 82 Patients in the Years 2002-2014. Biomed Res Int. 2015.

S173. D. Wang L, S.,Cheng, Z.,Xiao, N.,Cotter, C.,Hwang, J.,Li, X.,Yin, S.,Wang, J.,Bai, L.,Zheng, Z.,Wang, S. Transmission Risk from Imported Plasmodium vivax Malaria in the China-Myanmar Border Region. Emerg Infect Dis. 2015.

S174. I. M. El Hassan S, A.,Alzahrani, M. H.,Alhakeem, R. F.,Alhelal, M.,Alhogail, A.,Alsheikh, A. A.,Assiri, A. M.,ElGamri, T. B.,Faragalla, I. A.,Al-Atas, M.,Akeel, M. A.,Bani, I.,Ageely, H. M.,BinSaeed, A. A.,Kyalo, D.,Noor, A. M.,Snow, R. W. Progress toward malaria elimination in Jazan Province, Kingdom of Saudi Arabia: 2000-2014. Malar J. 2015.

S175. J. Feng X, H. H.,Zhang, L.,Yan, H.,Feng, X. Y.,Fang, W.,Xia, Z. G. The Plasmodium vivax in China: decreased in local cases but increased imported cases from Southeast Asia and Africa. Scientific Reports. 2015.

S176. K. A. Kanani A, Z. S.,Alkhatib, R.,Shadfan, B.,Al-Rashadan, M.,Hani, R. B. A retrospective study on imported malaria in Jordan. 2. Malaria among non-military Jordanians. Bull Soc Pathol Exot. 2015.

S177. M. E. Castellanos D, S.,Parsons, E.,Peruski, L. F.,Enríquez, F.,Ramírez, J. L.,Padilla, N. First imported Plasmodium ovale malaria in Central America: case report of a Guatemalan soldier and a call to improve its accurate diagnosis. Mil Med Res. 2015.

S178. M. Fernández López RG, J. M.,San Martín López, J. V.,Jaquetti, J.,García Arata, I.,Jiménez Navarro, C.,Cabello Clotet, N. Imported malaria including HIV and pregnant woman risk groups: overview of the case of a Spanish city 2004-2014. Malar J. 2015.

S179. M. Tseroni B, A.,Kapizioni, C.,Snounou, G.,Tsiodras, S.,Charvalakou, M.,Georgitsou, M.,Panoutsakou, M.,Psinaki, I.,Tsoromokou, M.,Karakitsos, G.,Pervanidou, D.,Vakali, A.,Mouchtouri, V.,Georgakopoulou, T.,Mamuris, Z.,Papadopoulos, N.,Koliopoulos, G.,Badieritakis, E.,Diamantopoulos, V.,Tsakris, A.,Kremastinou, J.,Hadjichristodoulou, C.,Malwest Project. Prevention of Malaria Resurgence in Greece through the Association of Mass Drug Administration (MDA) to Immigrants from Malaria-Endemic Regions and Standard Control Measures. Plos Neglected Tropical Diseases. 2015.

S180. P. Dharmawardena P, R. G.,Gunasekera, W. M.,Hewawitarane, M.,Mendis, K.,Fernando, D. Characterization of imported malaria, the largest threat to sustained malaria elimination from Sri Lanka. Malar J. 2015.

S181. R. H. Behrens N, P. E.,Jones, C. O. Imported malaria among people who travel to visit friends and relatives: is current UK policy effective or does it need a strategic change? Malar J. 2015.

S182. S. F. O'Brien D, G.,Seed, C. R.,Pillonel, J.,Fabra, C. C.,Davison, K.,Kitchen, A.,Steele, W. R.,Leiby, D. A. The Epidemiology of Imported Malaria and Transfusion Policy in 5 Nonendemic Countries. Transfus Med Rev. 2015.

S183. A. M. A. Alshahrani TMS, I.Al-Akhshami, A.Al-Ghamdi, M.Al-Zahrani, M. H.El Hassan, I.Kyalo, D.Snow, R. W. The changing malaria landscape in Aseer region, Kingdom of Saudi Arabia: 2000-2015. Malaria Journal. 2016.

S184. A. Oyinade S, K. Review and modelling of malaria crude incidence rate in a low incidence population, Illinois 1990 to 2013. Pan Afr Med J. 2016.

S185. B. C. Francis G, X.,Duggineni, S.,Thomas, J. M.,NicFhogartaigh, C.,Babiker, Z. O. Epidemiology and clinical features of imported malaria in East London. J Travel Med. 2016.

S186. C. Xu W, Q. K.,Li, J.,Xiao, T.,Yin, K.,Zhao, C. L.,Wang, Y. B.,Kong, X. L.,Zhao, G. H.,Sun, H.,Liu, X.,Huang, B. C. Characteristics of Imported Malaria and Species of Plasmodium Involved in Shandong Province, China (2012-2014). Korean J Parasitol. 2016.

S187. E. Casalino E, A.,Mentré, F.,Houzé, S. Hospitalization and ambulatory care in imported-malaria: evaluation of trends and impact on mortality. A prospective multicentric 14-year observational study. Malar J. 2016.

S188. E. Dirlikov R, C.,Morales, S.,Martínez, L. C.,Mendez, J. B.,Sanchez, A. C.,Burgos, J. H.,Santiago, Z.,Cuevas-Ruis, R. I.,Camacho, S. A.,Mercado, E. R.,Guzmán, J. F.,Ryff, K.,Luna-Pinto, C.,Arguin, P. M.,Chenet, S. M.,Silva-Flannery, L.,Ljolje, D.,Velázquez, J. C.,Thomas, D.,Garcia, B. R. Notes from the Field: Imported Cases of Malaria--Puerto Rico, July-October 2015. MMWR Morb Mortal Wkly Rep. 2016.

S189. E. T. Piperaki D, G. L. Malaria in Europe: emerging threat or minor nuisance? Clin Microbiol Infect. 2016.

S190. J. A. A. do Nascimento G, L. M.,Carvalho-Costa, F. A. Malaria epidemiology in the State of Piaui, Northeastern Brazil: a retrospective study with secondary data. Revista Da Sociedade Brasileira De Medicina Tropical. 2016.

S191. J. Poluga M, I.,Jordovic, J.,Dakic, Z.,Lavadinovic, L.,Stevanovic, G.,Milosevic, B.,Jevtovic, D.,Pavlovic, M. Clinical characteristics of imported malaria: An 11-year experience in a Serbian referral center. J Infect Dev Ctries. 2016.

S192. L. L. Li YX, G. J.Yu, T.Zou, Y.Wu, X. H.Zhong, B. Epidemiological analysis of malaria prevalence in Sichuan Province, 2012-2014. Chinese Journal of Schistosomiasis Control. 2016.

S193. M. El-Malky A, M. S.,Elsendiony, A.,Al-Harthi, S. A.,Zaghloul, D.,Salah, N. Imported Malaria To Makkah District, Saudi Arabia: Is There Any Risk Of Local Transmission? J Egypt Soc Parasitol. 2016.

S194. P. E. Neave H, A. E.,Gibney, K. B.,Leder, K. Imported infections: What information should be collected by surveillance systems to inform public health policy? Travel Medicine and Infectious Disease. 2016.

S195. S. D. Fernando D, P.,Semege, S.,Epasinghe, G.,Senanayake, N.,Rodrigo, C.,Premaratne, R. The risk of imported malaria in security forces personnel returning from overseas missions in the context of prevention of re-introduction of malaria to Sri Lanka. Malar J. 2016.

S196. S. L. Yeruva S, A.,Sarraf-Yazdy, M.,Gajjala, J. Imported Malaria over Fifteen Years in an Inner City Teaching Hospital of Washington DC. Korean J Parasitol. 2016.

S197. S. Li Y, S.,Wang, J.,Li, X.,Feng, J. Shifting from control to elimination: analysis of malaria epidemiological characteristics in Tengchong County around China-Myanmar border, 2005-2014. Malar J. 2016.

S198. X. Y. Y. Wang LLJ, T.Zhang, B. Y.Wang, S. Q.Wu, X. F.Wang, T. Y.Li, Y. L.Liu, M.Peng, Q. B.Zhang, W. H. Effects of a malaria elimination program: a retrospective study of 623 cases from 2008 to 2013 in a Chinese county hospital near the China - Myanmar border. Emerging Microbes & Infections. 2016.

S199. A. Dewanee Ranaweera D, M. N.,Pahalagedera, K.,de, A. W. Gunasekera W. M.,Dharmawardena, P.,Mak, K. W.,Wong, P. J.,Li, M. I.,Tan, C. H.,Hapuarachchi, H. C.,Herath, H. D.,Fernando, D. Diagnostic challenges and case management of the first imported case of Plasmodium knowlesi in Sri Lanka. Malar J. 2017.

S200. A. J. Tatem J, Peng,Ordanovich, Dariya,Falkner, Michael,Huang, Zhuojie,Howes, Rosalind,Hay, Simon I,Gething, Peter W,Smith, David L. The geography of imported malaria to non-endemic countries: a meta-analysis of nationally reported statistics. The Lancet Infectious Diseases. 2017.

S201. B. de Gier S, F. S.,Croughs, M.,van Genderen, P. J.,Keuter, M.,Visser, L. G.,van Vugt, M.,Sonder, G. J. Increase in imported malaria in the Netherlands in asylum seekers and VFR travellers. Malar J. 2017.

S202. B. S. Sánchez T, L. M. P.,Martín, S. G.,Pérez, E.,Grasa, C.,Valderrama, S.,Augusto, I.,Sierra, M.,Ros, M. G.,Aguado, I.,Hortelano, M. G. L. Imported malaria in children in Madrid, Spain, 2007-2013. Enferm Infecc Microbiol Clin. 2017.

S203. C. Ponce K, F.,Perpoint, T.,Miailhes, P.,Sigal, A.,Javouhey, E.,Gillet, Y.,Jacquin, L.,Douplat, M.,Tazarourte, K.,Potinet, V.,Simon, B.,Lavoignat, A.,Bonnot, G.,Sow, F.,Bienvenu, A. L.,Picot, S. Diagnostic accuracy of loop-mediated isothermal amplification (LAMP) for screening patients with imported malaria in a non-endemic setting. Parasite. 2017.

S204. C. Z. Yang HZ, R.Qian, D.Liu, Y.Zhao, Y.Li, S.Xu, B. Polymorphisms of Plasmodium falciparum k13-propeller gene among migrant workers returning to Henan Province, China from Africa. BMC Infectious Diseases. 2017.

S205. D. Luise D, D.,Visentin, F.,Marini, G.,Giaquinto, C.,Cattelan, A. Comparing imported malaria in adults and children presenting to an Italian teaching hospital: A 10-year retrospective study. Travel Med Infect Dis. 2017.

S206. E. Rees S-C, M.,Usdin, M.,Anderson, C.,Freedman, J.,de Burgh, J.,Kirkbride, H.,Chiodini, P.,Smith, V.,Blaze, M.,Whitty, C. J. M.,Balasegaram, S. Trend analysis of imported malaria in London; observational study 2000 to 2014. Travel Med Infect Dis. 2017.

S207. F. F. Norman L-P, A.,Salvador, F.,Treviño, B.,Calabuig, E.,Torrús, D.,Soriano-Arandes, A.,Ruíz-Giardín, J. M.,Monge-Maillo, B.,Pérez-Molina, J. A.,Perez-Ayala, A.,García, M.,Rodríguez, A.,Martínez-Serrano, M.,Zubero, M.,López-Vélez, R. Imported malaria in Spain (2009-2016): results from the +REDIVI Collaborative Network. Malar J. 2017.

S208. H. M. Wu F, Z. Q.,Zhao, D.,Chen, Y. L.,Liu, C. G.,Liang, X. A study on the epidemiological characteristics and infectious forecast model of malaria at Guangzhou Airport among Chinese returnees from Africa. Malar J. 2017.

S209. H. S. Akselrod MP, D.Simon, G. Imported malaria in travelers presenting to a tertiary urban hospital, 2000-2016. Open Forum Infectious Diseases. 2017.

S210. J. A. L.-P. Perez-Molina AT, B.Molina, I.Goikoetxea, J.Diaz-Menendez, M.Torrus, D.Calabuig, E.Benito, A.Lopez-Velez, R.Redivi Study, Grp. 6-year review of plus Redivi: a prospective registry of imported infectious diseases in Spain. Journal of Travel Medicine. 2017.

S211. M. Kimura K, M.,Hasegawa, C.,Mutoh, Y.,Kato, Y.,Maruyama, H. Imported malaria in pregnant women experienced in Japan. J Infect Chemother. 2017.

S212. N. Tejedor-Garavito D, N.,Pindolia, D.,Soble, A.,Ruktanonchai, N. W.,Alegana, V.,Le Menach, A.,Ntshalintshali, N.,Dlamini, B.,Smith, D. L.,Tatem, A. J.,Kunene, S. Travel patterns and demographic characteristics of malaria cases in Swaziland, 2010-2014. Malar J. 2017.

S213. P. Dharmawardena R, C.,Mendis, K.,de, A. W. Gunasekera Wmkt,Premaratne, R.,Ringwald, P.,Fernando, D. Response of imported malaria patients to antimalarial medicines in Sri Lanka following malaria elimination. PLoS One. 2017.

S214. P. K. Sriwichai SS, Y.Kiattibutr, K.Sirichaisinthop, J.Mueller, I.Cui, L. W.Sattabongkot, J. Imported Plasmodium falciparum and locally transmitted Plasmodium vivax: cross-border malaria transmission scenario in northwestern Thailand. Malaria Journal. 2017.

S215. P. Mornand V, C.,Minodier, P.,Faye, A.,Thellier, M.,Imbert, P. Severe imported malaria in children in France. A national retrospective study from 1996 to 2005. PLoS One. 2017.

S216. R. Dell'Acqua F, C.,Di Gennaro, F.,Lo Caputo, S.,Saracino, A.,Menegon, M.,L'Episcopia, M.,Severini, C.,Monno, L.,Castelli, F.,Angarano, G. An intricate case of multidrug resistant Plasmodium falciparum isolate imported from Cambodia. Malar J. 2017.

S217. R. Ruas P, A.,Nuak, J.,Sarmento, A.,Abreu, C. Non-falciparum malaria imported mainly from Africa: a review from a Portuguese hospital. Malar J. 2017.

S218. S. D. Fernando B, R.,Dharmawardena, P.,Harintheran, A.,Raviraj, K.,Rodrigo, C.,Danansuriya, M.,Wickremasinghe, R. The need for preventive and curative services for malaria when the military is deployed in endemic overseas territories: a case study and lessons learned. Military Medical Research. 2017.

S219. S. M. Chenet S-F, L.,Lucchi, N. W.,Dragan, L.,Dirlikov, E.,Mace, K.,Rivera-García, B.,Arguin, P. M.,Udhayakumar, V. Molecular Characterization of a Cluster of Imported Malaria Cases in Puerto Rico. Am J Trop Med Hyg. 2017.

S220. A. Calderaro P, G.,Montecchini, S.,Buttrini, M.,Rossi, S.,Dell'Anna, M. L.,De Remigis, V.,Arcangeletti, M. C.,Chezzi, C.,De Conto, F. High prevalence of malaria in a non-endemic setting: comparison of diagnostic tools and patient outcome during a four-year survey (2013-2017). Malar J. 2018.

S221. A. J. DePina N, E. H. A.,Barbosa Andrade, A. J.,Dia, A. K.,Moreira, A.,Faye, O.,Seck, I. Achievement of malaria pre-elimination in Cape Verde according to the data collected from 2010 to 2016. Malar J. 2018.

S222. A. Malede A, K.,Aemero, M.,Robele, S.,Kloos, H. Travel to farms in the lowlands and inadequate malaria information significantly predict malaria in villages around Lake Tana, northwest Ethiopia: a matched case-control study. Malar J. 2018.

S223. D. Ranaweera KR, R. M. J.,Silva, P.,Hettiarachchi, R.,Gunasekera, Wmktaw,Herath, H.,Fernando, D. Severe Plasmodium vivax malaria, HIV, tuberculosis co-infection in a Sri Lankan traveller: case management and challenges during the prevention of malaria reintroduction phase. Malar J. 2018.

S224. E. Farag B, D.,Chehab, M. A. H.,Al-Dahshan, A.,Bala, M.,Ganesan, N.,Al Abdulla, Y. A.,Al Thani, M.,Sultan, A. A.,Al-Romaihi, H. Epidemiology of Malaria in the State of Qatar, 2008-2015. Mediterr J Hematol Infect Dis. 2018.

S225. E. H. Lee M, R. H.,Masuoka, P.,Schiffman, E.,Wanduragala, D. M.,DeFraites, R.,Dunlop, S. J.,Stauffer, W. M.,Hickey, P. W. Predicting Risk of Imported Disease with Demographics: Geospatial Analysis of Imported Malaria in Minnesota, 2010-2014. Am J Trop Med Hyg. 2018.

S226. F. Pagès H, S.,Kurtkowiak, B.,Balleydier, E.,Chieze, F.,Filleul, L. Status of imported malaria on Réunion Island in 2016. Malar J. 2018.

S227. G. Ding Z, G.,Cao, C.,Miao, P.,Cao, Y.,Wang, W.,Gu, Y.,Xu, S.,Wang, S.,Zhou, H.,Cao, J. The challenge of maintaining microscopist capacity at basic levels for malaria elimination in Jiangsu Province, China. BMC Public Health. 2018.

S228. G. Froeschl N, H. D.,von Sonnenburg, F.,Bretzel, G.,Polanetz, R.,Kroidl, I.,Seilmaier, M.,Orth, H. M.,Jordan, S.,Kremsner, P.,Vygen-Bonnet, S.,Pritsch, M.,Hoelscher, M.,Rothe, C. Retrospective clinical case series study in 2017 identifies Plasmodiumknowlesi as most frequent Plasmodium species in returning travellers from Thailand to Germany. Eurosurveillance. 2018.

S229. H. Akselrod S, M. J.,Zheng, Z. N.,Keiser, J.,Parenti, D. M.,Simon, G. L. Characteristics and Severity of Disease among 100 Cases of Imported Malaria Seen at a US University Hospital, 2000-2017. American Journal of Tropical Medicine and Hygiene. 2018.

S230. H. Bastaki M, L.,Cassell, J.,Rait, G. Imported malaria in the UK, 2005 to 2016: Estimates from primary care electronic health records. PLoS One. 2018.

S231. H. Hiwat M-L, B.,Cairo, H.,Hardjopawiro, L.,Boerleider, A.,Duarte, E. C.,Yadon, Z. E. Malaria epidemiology in Suriname from 2000 to 2016: trends, opportunities and challenges for elimination. Malar J. 2018.

S232. I. G. Rainova H, R. N.,Kaftandjiev, I. T.,Mikov, O. D.,Tsvetkova, N. D. Imported malaria in Bulgaria, status and prognosis after eradication in 1965. J Infect Public Health. 2018.

S233. J. Xia H, X.,Sun, L.,Zhu, H.,Lin, W.,Dong, X.,Wu, D.,Qiu, J.,Zheng, L.,Cao, M.,Liu, S.,Zhang, H. Epidemiological characteristics of malaria from control to elimination in Hubei Province, China, 2005-2016. Malar J. 2018.

S234. K. E. Mace A, P. M.,Tan, K. R. Malaria Surveillance - United States, 2015. MMWR Surveill Summ. 2018.

S235. K. R. Rijal A, B. A.,Ghimire, P.,Banjara, M. R.,Hanboonkunupakarn, B.,Imwong, M.,Chotivanich, K.,Acharya, B.,Lal, V. K.,Thakur, G. D.,Day, N. P. J.,White, N. J.,Pukrittayakamee, S. Epidemiology of plasmodium vivax malaria infection in Nepal. American Journal of Tropical Medicine and Hygiene. 2018.

S236. L. E. B. N. Nabarro DB, C.Nadjm, B.Smith, V.Blaze, M.Checkley, A. M.Chiodini, P. L.Sutherland, C. J.Whitty, C. J. M. Geographical and temporal trends and seasonal relapse in Plasmodium ovale spp. and Plasmodium malariae infections imported to the UK between 1987 and 2015. Bmc Medicine. 2018.

S237. M. Develoux LL, G.,Lafon-Desmurs, B.,Magne, D.,Belkadi, G.,Daray, E.,Pialoux, G.,Hennequin, C. Imported malaria in pregnant women: report from a French University Centre. Infection. 2018.

S238. M. H. Abdelraheem B, D.,Idris, M. A.,Mukhtar, M. M.,Hamid, M. M. A.,Imam, Z. S.,Getachew, S.,Sehgal, R.,Kaur, H.,Gadalla, A. H.,Al-Hamidhi, S.,Al-Hashami, Z.,Al-Jabri, A.,Sultan, A. A.,Babiker, H. A. Genetic diversity and transmissibility of imported Plasmodium vivax in Qatar and three countries of origin. Sci Rep. 2018.

S239. M. H. Al Zahrani O, A. I.,Abdoon, A. M. O.,Ibrahim, A. A.,Alhogail, A.,Elmubarak, M.,Elamin, Y. E.,AlHelal, M. A.,Alshahrani, A. M.,Abdelgader, T. M.,Saeed, I.,El Gamri, T. B.,Alattas, M. S.,Dahlan, A. A.,Assiri, A. M.,Maina, J.,Li, X. H.,Snow, R. W. Cross-border movement, economic development and malaria elimination in the Kingdom of Saudi Arabia. BMC Med. 2018.

S240. N. Dlamini Z, Z.,Kunene, S.,Geoffroy, E.,Ntshalintshali, N.,Owiti, P.,Sikhondze, W.,Makadzange, K.,Zachariah, R. From diagnosis to case investigation for malaria elimination in Swaziland: is reporting and response timely? Public Health Action. 2018.

S241. P. Stano A, A.,Merelli, M.,Mascarello, M.,Maurel, C.,Avolio, M.,Bassetti, M.,De Rosa, R.,Luzzati, R.,Modolo, M. L.,Scarparo, C.,Camporese, A. Epidemiological and clinical features of imported malaria at the three main hospitals of the Friuli-Venezia Giulia Region, Italy. Infect Dis Health. 2018.

S242. R. Chu Z, X.,Xu, S.,Chen, L.,Tang, J.,Li, Y.,Chen, J.,Xuan, Y.,Zhu, G.,Cao, J.,Cheng, Y. Limited genetic diversity of N-terminal of merozoite surface protein-1 (MSP-1) in Plasmodium ovale curtisi and P. ovale wallikeri imported from Africa to China. Parasit Vectors. 2018.

S243. S. Antinori N, M.,Grande, R.,Passerini, S.,Ridolfo, A. L.,Galimberti, L.,Oreni, L.,Schinaia, S.,Milazzo, L.,Galli, M.,Corbellino, M.,Gismondo, M. R.,Zanchetta, N.,Cordier, L.,Rizzardini, G. Epidemiological and clinical characteristics of imported malaria in adults in Milan, Italy, 2010–2015. European Journal of Internal Medicine. 2018.

S244. S. Vygen-Bonnet S, K. Changes in malaria epidemiology in Germany, 2001-2016: a time series analysis. Malar J. 2018.

S245. Y. Yang L, Y.,Xie, Z.,Wu, S.,Yang, L.,Li, W.,Quan, X. Epidemiology of Malaria in Yulin, South China 1999-2016: Imported Malaria Threatens Zero Local Case Status. Vector-Borne and Zoonotic Diseases. 2018.

S246. A. Baranova S, V.,Morozova, L.,Turbabina, N.,Morozov, E. Imported Plasmodium vivax Malaria in the Russian Federation from Western Sub-Saharan Africa. J Trop Med. 2019.

S247. A. Izri C, S.,Leblanc, C.,Cohen, Y.,Bouchaud, O.,Durand, R. Plasmodium vivax severe imported malaria in two migrants in France. Malar J. 2019.

S248. A. J. Rodríguez-Morales S, J. A.,Risquez, A.,Villamil-Gómez, W. E.,Paniz-Mondolfi, A. Consequences of Venezuela's massive migration crisis on imported malaria in Colombia, 2016-2018. Travel Med Infect Dis. 2019.

S249. A. Wångdahl W, K.,Saduddin, D.,Bottai, M.,Ydring, E.,Vikerfors, T.,Färnert, A. Severity of Plasmodium falciparum and Non-falciparum Malaria in Travelers and Migrants: A Nationwide Observational Study Over 2 Decades in Sweden. J Infect Dis. 2019.

S250. B. C. Carlos R, L. D. P.,Christophides, G. K.,Souza-Neto, J. A. A comprehensive analysis of malaria transmission in Brazil. Pathogens and Global Health. 2019.

S251. D. Bansal B, P. K.,Acharya, A.,Abdelraheem, M. H.,Patel, P.,Elmalik, A.,Abosalah, S.,Khan, F. Y.,ElKhalifa, M.,Kaur, H.,Farag, E.,Sarmah, N. P.,Mohapatra, P. K.,Sehgal, R.,Mahanta, J.,Sultan, A. A. Molecular surveillance of putative drug resistance markers of antifolate and artemisinin among imported Plasmodium falciparum in Qatar. Pathog Glob Health. 2019.

S252. D. Khuu E, M. L.,Bristow, B. N.,Javanbakht, M.,Ash, L. R.,Shafir, S. C.,Sorvillo, F. J. Economic impact of malaria-related hospitalizations in the United States, 2000–2014. Journal of Infection and Public Health. 2019.

S253. E. Jamshidi EA, H.,Yousefi-Nooraie, R.,Raeisi, A.,Malekafzali Ardakani, H.,Sadeghi, R.,Hanafi-Bojd, A. A.,Majdzadeh, R. A social network analysis on immigrants and refugees access to services in the malaria elimination context. Malaria Journal. 2019.

S254. E. Kendjo H, S.,Mouri, O.,Taieb, A.,Gay, F.,Jauréguiberry, S.,Tantaoui, I.,Ndour, P. A.,Buffet, P.,Piarroux, M.,Thellier, M.,Piarroux, R. Epidemiologic Trends in Malaria Incidence Among Travelers Returning to Metropolitan France, 1996-2016. JAMA Netw Open. 2019.

S255. F. Panin O, E.,Galli, L.,De Martino, M.,Chiappini, E. Increasing imported malaria in children and adults in Tuscany, Italy, (2000 to 2017): A retrospective analysis. Travel Med Infect Dis. 2019.

S256. H. G. Albuquerque P, P. C.,Toledo, L. M.,Sabroza, P. C.,Pereira, R. D. S.,Caldas, J. P.,Angelo, J. R.,Dias, C. G.,Suárez-Mutis, M. C. Imported malaria in Rio de Janeiro state between 2007 and 2015: an epidemiologic approach. Mem Inst Oswaldo Cruz. 2019.

S257. H. H. Chang W, A.,Sinha, I.,Jacob, C. G.,Mahmud, A.,Uddin, D.,Zaman, S. I.,Hossain, M. A.,Faiz, M. A.,Ghose, A.,Sayeed, A. A.,Rahman, M. R.,Islam, A.,Karim, M. J.,Rezwan, M. K.,Shamsuzzaman, A. K. M.,Jhora, S. T.,Aktaruzzaman, M. M.,Drury, E.,Gonçalves, S.,Kekre, M.,Dhorda, M.,Vongpromek, R.,Miotto, O.,Engø-Monsen, K.,Kwiatkowski, D.,Maude, R. J.,Buckee, C. Mapping imported malaria in Bangladesh using parasite genetic and human mobility data. Elife. 2019.

S258. H. Sun L, J.,Xu, C.,Xiao, T.,Wang, L.,Kong, X.,Wang, Y.,Zhang, B.,Zhao, C.,Huang, B.,Wei, Q. Increasing number of imported Plasmodium ovale wallikeri malaria in Shandong Province, China, 2015-2017. Acta Trop. 2019.

S259. K. Sondén R, T.,Wångdahl, A.,Ydring, E.,Vygen-Bonnet, S.,Kobbe, R.,Douhan, J.,Hammar, U.,Duijster, J.,de Gier, B.,Freedman, J.,Gysin, N.,Stark, K.,Stevens, F.,Vestergaard, L. S.,Tegnell, A.,Färnert, A. Malaria in Eritrean migrants newly arrived in seven European countries, 2011 to 2016. Eurosurveillance. 2019.

S260. M. A. Karim K, M. M.,Siddiqui, M. A.,Laskar, M. S. I.,Saha, A.,Naher, S. Epidemiology of Imported Malaria in Netrokona District of Bangladesh 2013-2018: Analysis of Surveillance Data. Malar Res Treat. 2019.

S261. M. Douine S, A.,Hiwat, H.,Briolant, S.,Nacher, M.,Belleoud, D.,Le Tourneau, F. M.,Bogreau, H.,De Laval, F. Investigation of a possible malaria epidemic in an illegal gold mine in French Guiana: An original approach in the remote Amazonian forest. Malaria Journal. 2019.

S262. P. Dharmawardena P, R.,Mendis, K.,Wickemasinghe, R.,Rodrigo, C.,Harintheran, A.,Fernando, D. Effectiveness of passive case detection for imported malaria in a hospital setting in Sri Lanka during the prevention of re-introduction phase of malaria. International Health. 2019.

S263. Q. M. Moyo B, M.,Lynn, R.,Lever, A. M. L. Persistence of Imported Malaria Into the United Kingdom: An Epidemiological Review of Risk Factors and At-risk Groups. Clinical Infectious Diseases. 2019.

S264. R. Jaramillo-Ochoa S, R.,Farrell, D. F.,Cueva-Aponte, C.,Beltran-Ayala, E.,Gonzaga, J. L.,Ordonez-Leon, T.,Quintana, F. A.,Ryan, S. J.,Stewart-Ibarra, A. M. Effects of Political Instability in Venezuela on Malaria Resurgence at Ecuador-Peru Border, 2018. Emerging Infectious Diseases. 2019.

S265. R. Zhou Y, C.,Li, S.,Zhao, Y.,Liu, Y.,Qian, D.,Wang, H.,Lu, D.,Zhang, H.,Huang, F. Molecular Surveillance of Drug Resistance of Plasmodium falciparum Isolates Imported from Angola in Henan Province, China. Antimicrob Agents Chemother. 2019.

S266. S. Khine K, N. T. T.,Thekkur, P.,Lin, Z.,Thi, A. Malaria hot spot along the foothills of Rakhine state, Myanmar: geospatial distribution of malaria cases in townships targeted for malaria elimination. Tropical Medicine and Health. 2019.

S267. S. Takaya K, Y.,Katanami, Y.,Yamamoto, K.,Kutsuna, S.,Takeshita, N.,Hayakawa, K.,Kanagawa, S.,Komaki-Yasuda, K.,Kano, S.,Ohmagari, N. Imported Malaria at a Referral Hospital in Tokyo from 2005 to 2016: Clinical Experience and Challenges in a Non-Endemic Setting. Am J Trop Med Hyg. 2019.

S268. T. Zhang X, X.,Jiang, J.,Yu, C.,Tian, C.,Xie, Q.,Li, W.,Monge-Maillo, B.,López-Vélez, R. Risk factors of severe imported malaria in Anhui province, China-//-Migration and malaria in europe. Acta Trop. 2019.

S269. V. M. Karunasena M, M.,Koo, C.,Amarasinghe, S.,Senaratne, A. S.,Hasantha, R.,Hewavitharana, M.,Hapuarachchi, H. C.,Herath, H. D. B.,Wickremasinghe, R.,Mendis, K. N.,Fernando, D.,Ranaweera, D. The first introduced malaria case reported from Sri Lanka after elimination: implications for preventing the re-introduction of malaria in recently eliminated countries. Malar J. 2019.

S270. Y. Hawash I, K.,Alsharif, K.,Alsanie, W. Malaria Prevalence in a Low Transmission Area, Jazan District of Southwestern Saudi Arabia. Korean J Parasitol. 2019.

S271. Z. Herrador F-M, B.,Quesada-Cubo, V.,Diaz-Garcia, O.,Cano, R.,Benito, A.,Gómez-Barroso, D. Imported cases of malaria in Spain: observational study using nationally reported statistics and surveillance data, 2002-2015. Malar J. 2019.

S272. A. Al-Rumhi A-H, Z. S.,Al-Hamidhi, S.,Gadalla, A.,Naeem, R.,Ranford-Cartwright, L.,Pain, A.,Sultan, A. A.,Babiker, H. A. Influx of diverse, drug resistant and transmissiblePlasmodium falciparuminto a malaria-free setting in Qatar. Bmc Infectious Diseases. 2020.

S273. A. J. DePina S, G.,Barros, H. S. B.,Moreira, A. L.,Dia, A. K.,Furtado, U. D.,Faye, O.,Seck, I.,Niang, E. H. A. Updates on malaria epidemiology and profile in Cabo Verde from 2010 to 2019: the goal of elimination. Malar J. 2020.

S274. D. F. L. Escobar NWA, R.Valenzuela, M. T.Udhayakumar, V.Jercic, M. I.Chenet, S. M. Molecular and epidemiological characterization of imported malaria cases in Chile. Malaria Journal. 2020.

S275. H. I. Shin K, B.,Kim, Y. J.,Kim, T. Y.,Cho, S. H.,Lee, S. E. Diagnosis and Molecular Analysis on Imported Plasmodium ovale curtisi and P. ovale wallikeri Malaria Cases from West and South Africa during 2013-2016. Korean J Parasitol. 2020.

S276. J. Louzada dA, N. C. V.,de Araujo, J. L. P.,Silva, J.,Carvalho, T. M.,Escalante, A. A.,Oliveira-Ferreira, J. The impact of imported malaria by gold miners in Roraima: characterizing the spatial dynamics of autochthonous and imported malaria in an urban region of Boa Vista. Mem Inst Oswaldo Cruz. 2020.

S277. J. Mischlinger R, C.,Álvarez-Martínez, M. J.,Bühler, S.,Paul, M.,Schlagenhauf, P.,Petersen, E.,Ramharter, M. Imported Malaria in Countries where Malaria Is Not Endemic: a Comparison of Semi-immune and Nonimmune Travelers. Clin Microbiol Rev. 2020.

S278. L. V. Lepore FDA, A.Grilli, E.Corpolongo, A.Scorzolini, L.Nisii, C.Calleri, G.Castelli, F.Chirianni, A.Ippolito, G.Nicastri, E. Clinical management of imported malaria in Italy: results from a national cross-sectional survey in 2015. The new microbiologica. 2020.

S279. L. Zekar S, T. Plasmodium Falciparum Malaria. 2020.

S280. L. Zhao P, L.,Qin, Y.,Lu, Y.,Zeng, W.,Xiang, Z.,Qin, P.,Chen, X.,Li, C.,Zhang, Y.,Wang, S.,Si, Y.,Yang, G.,Rosenthal, B. M.,Huang, Y.,Yang, Z. Widespread resistance mutations to sulfadoxine-pyrimethamine in malaria parasites imported to China from Central and Western Africa. Int J Parasitol Drugs Drug Resist. 2020.

S281. M. Thellier S, F.,Musset, L.,Cot, M.,Velut, G.,Kendjo, E.,Pradines, B. Changes in malaria epidemiology in France and worldwide, 2000-2015. Med Mal Infect. 2020.

S282. P. K. R. Bharti HN, S.Jayswar, H.Saha, K. B.Shukla, M. M.Mishra, A. K.Sharma, R. K.Das, A.Kaur, H.Wattal, S. L.Lal, A. A. Demonstration of indigenous malaria elimination through Track-Test-Treat-Track (T4) strategy in a Malaria Elimination Demonstration Project in Mandla, Madhya Pradesh. Malaria Journal. 2020.
